# Uncertainty-Informed Structural Evaluation of a Halo-Gravity Traction Mobility System

**DOI:** 10.3390/bioengineering13091048

**Published:** 2026-09-09

**Authors:** Aylara Ölçmen, Jennifer Baggett, Sameer B. Mulani, Semih M. Ölçmen, Easir Arafat Papon

**Affiliations:** 1 STERIS Corporation, Birmingham, AL 35222, USA; aylaraolcmen@gmail.com; 2 Department of Biomedical Engineering, The University of Alabama at Birmingham, Birmingham, AL 35294, USA; 3 Department of Aerospace Engineering and Mechanics, The University of Alabama, Tuscaloosa, AL 35487, USA; jlbaggett@ua.edu (J.B.); sameer.mulani@ua.edu (S.B.M.); mpapon@eng.ua.edu (E.A.P.)

**Keywords:** halo-gravity traction, uncertainty quantification, polynomial chaos expansion, tipping stability, reduced-order modeling, structural screening

## Abstract

Halo-gravity traction (HGT) is widely used as a preoperative treatment for severe pediatric spinal deformities by gradually applying traction forces through a cranial fixation system. Although the clinical effectiveness of HGT has been extensively documented, uncertainty associated with the structural response and operational behavior of mobile HGT systems has received comparatively little attention. This study presents a preliminary coupled clinical–structural uncertainty quantification framework for engineering evaluation of a mobile halo-gravity traction wheelchair. Reduced-order structural and clinical surrogate models are combined with Latin Hypercube Sampling, bounded beta-distributed input variables, third-order Polynomial Chaos Expansion, and Sobol sensitivity analysis. Sixteen uncertain patient, operational, geometric, fabrication, and halo-interface parameters are propagated through models of combined beam–column loading, elastic buckling, welded-joint loading, scenario-based wheelchair tipping stability, halo-pin load concentration, traction delivery, and representative clinical response. Thirty-two alternative beta-distribution shape cases are examined to assess sensitivity to the assumed marginal distribution shapes. Within the investigated bounds and reduced-order modeling assumptions, the computational screening model did not predict loss of the prescribed structural margins. The results identify the parameters governing the different response modes and demonstrate how uncertainty in mobility-related acceleration, support geometry, traction loading, fabrication efficiency, and halo-pin load transfer can be evaluated within a unified framework. Because the model has not yet been validated through dedicated prototype testing or higher-fidelity full-system simulation, the results should be interpreted as uncertainty-informed engineering screening rather than as experimental verification or clinical certification of device safety.

## 1. Introduction

### 1.1. Uncertainty Quantification in Biomedical Engineering

Finite element analysis (FEA) has become an established tool in computational biomechanics for the design and evaluation of orthopedic implants and medical devices, including hip and knee replacements, spinal fixation systems, fracture fixation devices, and dental implants. Numerous studies have employed FEA to evaluate implant stresses, optimize device geometry, improve fatigue performance, and develop patient-specific treatment strategies. More recently, uncertainty quantification (UQ) has become increasingly important in computational biomechanics and medical device evaluation by accounting for variability in material properties, anatomical geometry, implant positioning, physiological loading, and other model parameters. These probabilistic approaches improve confidence in computational predictions and provide a more rigorous assessment of device performance than traditional deterministic analyses [1,2,3,4].

Recent research has consequently shifted from deterministic finite element analyses toward probabilistic computational frameworks capable of propagating uncertainties through biomechanical models. Monte Carlo simulation, Polynomial Chaos Expansion (PCE), and verification, validation, and uncertainty quantification (VVUQ) methodologies have been successfully applied to orthopedic biomechanics, patient-specific analyses, spinal fixation systems, cardiovascular devices, and other implantable medical systems. These approaches enable engineers to quantify the influence of uncertain design variables on stresses, strains, fatigue life, and structural reliability while providing a more realistic assessment of device performance than deterministic methods alone.

Among the available uncertainty propagation techniques, Polynomial Chaos Expansion (PCE) has emerged as one of the most effective surrogate modeling approaches because it represents stochastic system responses using orthogonal polynomial basis functions defined over the probability space of uncertain input variables. Since the generalized polynomial chaos framework introduced by Xiu and Karniadakis [5,6], considerable research has focused on improving the efficiency, scalability, and robustness of PCE for practical engineering applications. These developments include adaptive and weighted least-squares formulations, basis adaptivity, sequential adaptive sampling, and classifier-based sparse expansions for high-dimensional stochastic systems [7,8,9], sensitivity-enhanced polynomial chaos methods for improved uncertainty propagation in nonlinear and chaotic systems [10,11], and efficient algorithms for dynamic systems, reliability analysis, and uncertainty-informed design optimization [12,13,14,15]. Collectively, these advances have substantially reduced the computational cost associated with uncertainty propagation while maintaining predictive accuracy comparable to conventional Monte Carlo simulation, making PCE an attractive methodology for engineering design, reliability assessment, sensitivity analysis, and uncertainty-informed decision-making.

Despite these advances, uncertainty-aware computational methods have been applied primarily to implantable orthopedic devices, cardiovascular systems, and patient-specific biomechanical analyses. Comparatively little attention has been devoted to orthopedic treatment systems whose primary purpose is the application of controlled therapeutic loads rather than permanent implantation. Halo-gravity traction represents one such application, where patient variability, loading uncertainty, and structural response interact to determine both treatment effectiveness and device safety.

### 1.2. Halo-Gravity Traction Systems

Halo-gravity traction (HGT) is a gradual correction technique used for patients with severe spinal deformities, particularly pediatric scoliosis and kyphoscoliosis. Controlled traction is applied through a halo ring secured to the skull, allowing the spine, surrounding soft tissues, ligaments, and musculoskeletal structures to deform progressively over a period of days or weeks before definitive corrective surgery. Beyond reducing spinal deformity, HGT improves surgical preparation, reduces the magnitude of acute intraoperative correction, and often improves respiratory function and nutritional status in medically fragile patients.

Clinical studies have demonstrated that HGT typically produces deformity corrections on the order of 25–40%, together with measurable improvements in pulmonary capacity and overall preoperative condition [16,17,18,19,20]. However, treatment outcomes exhibit substantial patient-to-patient variability because correction depends on factors including initial deformity severity, patient anatomy, spinal flexibility, traction-force progression, treatment duration, and pin placement. Furthermore, the spine and surrounding tissues exhibit time-dependent viscoelastic behavior, so correction develops gradually through tissue creep, ligament deformation, muscle relaxation, and structural adaptation under sustained loading rather than as an instantaneous elastic response [17,21].

The mechanical system used to deliver halo-gravity traction also contributes significantly to treatment performance. Conventional HGT systems commonly employ suspended weights, pulley mechanisms, hospital beds, wheelchairs, and manually assembled support structures. While clinically effective, these systems introduce engineering uncertainties associated with pulley friction, structural flexibility, caregiver handling, patient motion, wheelchair acceleration, and load transfer through the halo ring [22,23,24]. As a result, the actual traction load delivered to the patient may differ from the prescribed load, while structural components experience uncertain loading conditions that influence safety and long-term durability.

The mobile halo-gravity traction wheelchair considered in the present study was originally conceived, designed, and prototyped through a senior biomedical engineering capstone project conducted at the University of Alabama at Birmingham. Rather than revisiting the device-design process itself, the present work builds upon the completed prototype to perform a comprehensive uncertainty-informed engineering evaluation. The system integrates a wheelchair, vertical support member, guided weight carriage, pulley-and-cable traction mechanism, welded rear support frame, anti-tip caster supports, and attachment hardware into a single structural system intended to improve traction delivery during patient mobility.

Because the device operates at the intersection of clinical treatment and structural mechanics, its engineering evaluation must consider traction delivery, combined structural loading, buckling resistance, weld integrity, scenario-based tipping stability, and halo-pin load transfer. These responses depend on uncertain patient characteristics, operational conditions, geometric parameters, and fabrication-related properties. A deterministic analysis based only on nominal inputs would therefore provide an incomplete representation of the possible system responses. The present study develops a coupled clinical–structural uncertainty quantification framework using Latin Hypercube Sampling (LHS), bounded beta-distributed inputs, Polynomial Chaos Expansion (PCE), and Sobol sensitivity analysis to propagate the prescribed uncertainties and identify the parameters governing the principal screening-level response measures.

The primary contribution of this work is the integration of clinically informed loading parameters with a reduced-order structural uncertainty model for a mobile HGT wheelchair system. Rather than treating the vertical support, welded frame, pulley and weight system, rear-caster support polygon, and halo-pin load transfer as unrelated components, the framework evaluates their interactions through traction-delivery, beam–column, buckling, weld, scenario-based tipping, and pin-load screening models. The resulting computational framework is intended to identify influential parameters, compare competing structural limit states, and guide subsequent model refinement, prototype testing, and higher-fidelity analysis. It is not intended to establish clinical efficacy or to provide experimental certification of device safety.

## 2. System Description and Structural Modeling

The halo-gravity traction (HGT) wheelchair considered in this study is a prototype mobile pediatric traction platform intended to support the preoperative treatment of severe scoliosis and kyphosis. The system combines a conventional wheelchair base with an integrated rear structural assembly that carries the guided traction-weight system and associated cable-and-pulley hardware. The primary load-carrying member is a vertical C-channel support welded to the rear frame of the wheelchair. A guided carriage travels along this member and supports the traction weights, while the pulley system redirects the applied load toward the halo ring. Rear-caster supports extend the effective support polygon and increase resistance to rearward tipping.

**Figure 1:**
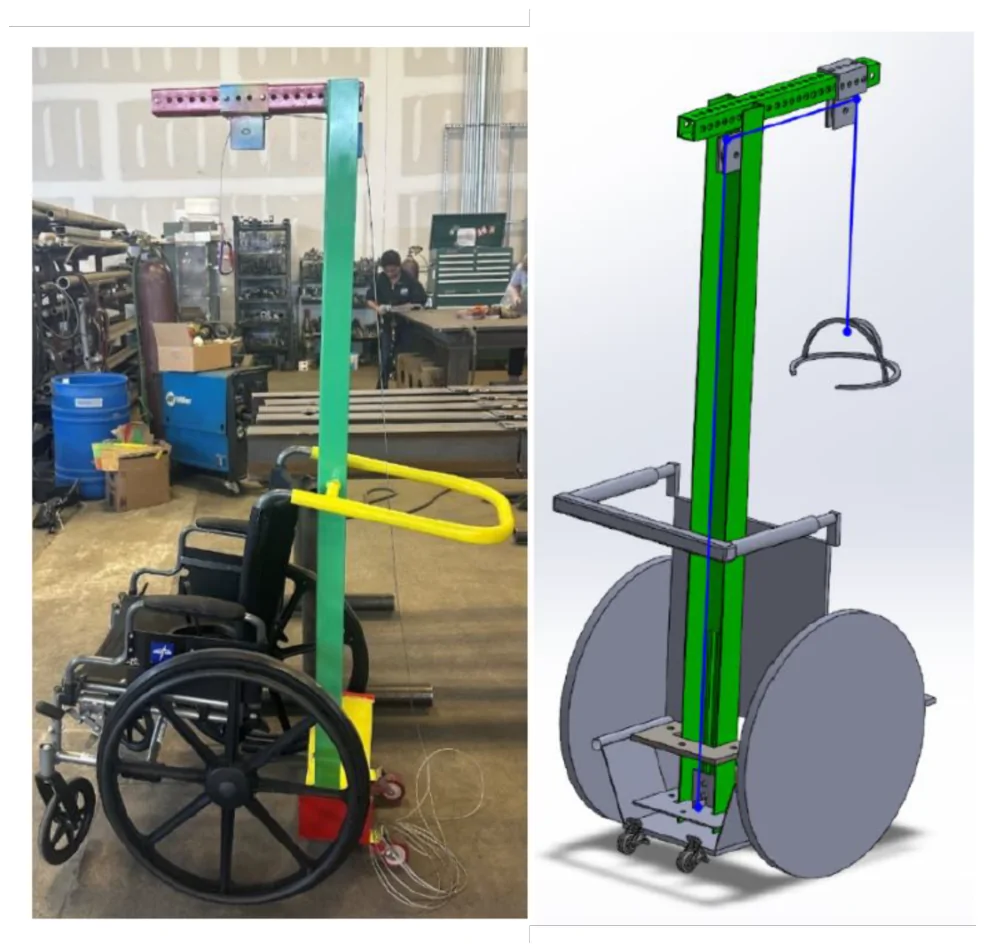
Fabricated prototype and corresponding CAD representation of the proposed halo-gravity traction wheelchair support system.

Figure 1 presents the fabricated prototype and the corresponding CAD representation used to define the geometry and load paths of the reduced-order model. The integrated rear support frame, guided vertical member, overhead traction assembly, and rear-caster supports are visible in both configurations. The prototype was developed to permit traction during patient transport while remaining compatible with the mobility and dimensional constraints of a conventional hospital wheelchair. The prototype and CAD model provide the geometric basis for the present computational screening analysis; they do not constitute experimental validation of the reduced-order structural model.

Because the system is intended for use in a hospital environment, its overall dimensions must remain compatible with patient rooms, hallways, elevators, and door openings. The principal dimensional constraints adopted during prototype development were

(1) $$W_{\mathrm{sys}}\leq 36\;\mathrm{in}.,$$

and

(2) $$H_{\mathrm{sys}}\leq 80\;\mathrm{in}.,$$

where $W_{\mathrm{sys}}$ and $H_{\mathrm{sys}}$ denote the overall system width and height, respectively. These dimensional limitations affect more than the external package size. They constrain the available base width, placement of the rear-caster supports, height of the vertical member, pulley location, and traction-line geometry. Consequently, the dimensional configuration influences both clinical usability and the structural and tipping responses of the system.

During treatment, the prescribed traction force is increased gradually toward a clinically selected loading level. The upper bound considered in the present model is

(3) $$F_{t,\max}=0.50W_{p},$$

where $W_{p}$ denotes patient body weight. The actual traction ratio is determined clinically according to factors such as patient tolerance, treatment stage, and deformity severity. Within the present engineering model, increased patient weight or traction ratio increases the cable tension, compressive loading of the support structure, halo-pin load, pulley reaction, and load transferred through the welded frame.

The structural load path begins at the halo ring, where the cable transmits the prescribed traction force. The cable tension is redirected by the pulley system and balanced by the guided weight carriage. The resulting reactions are transferred through the vertical support member, welded rear frame, wheelchair-attachment points, rear-caster supports, and ultimately to the ground. This idealized load path was inferred from the prototype geometry and mechanical arrangement and forms the basis of the reduced-order model. Because dedicated full-system load testing has not yet been performed, the assumed distribution of reactions among these components remains a modeling approximation requiring future experimental validation.

For the member-level analysis, the vertical support is conservatively idealized as a fixed–free cantilever beam–column. The traction-related load is transferred through a short horizontal arm at a finite eccentricity from the centroidal axis of the vertical member. Consequently, the member is modeled as experiencing simultaneous axial compression, first-order eccentric bending, and second-order geometric amplification (*P*–Δ), rather than pure concentric column compression. This idealization represents the dominant load-transfer mechanisms while retaining the computational efficiency required for the uncertainty analysis.

The principal mechanisms represented in the reduced-order model include cable and traction loading, compression and eccentric bending of the vertical support, global elastic buckling, welded-joint loading, forward, lateral, and rearward tipping stability, halo-pin load concentration, and mobility-related inertial loading. Because the traction-weight carriage is mechanically guided, large pendulum-type oscillations associated with freely suspended weights are reduced. Nevertheless, wheelchair acceleration, braking, turning, floor transitions, patient movement, and geometric misalignment may introduce additional inertial loads and secondary moments. These effects are represented through bounded operational and geometric inputs rather than through a full transient multibody model.

The prescribed traction force is defined as

(4) $$F_{t}=r_{t}W_{p},$$

where $r_{t}$ is the traction-to-body-weight ratio. Accounting for the pulley efficiency $\eta_{p}$, the guided weight-stack load is

(5) $$F_{w}=\frac{F_{t}}{\eta_{p}}.$$

The adverse vertical inertial load associated with acceleration or deceleration of the guided weight carriage is approximated as

(6) $$F_{d,v}=F_{w}a_{g,v},$$

where $a_{g,v}=a_{v}/g$ is the adverse vertical carriage acceleration normalized by gravitational acceleration. This sampled term is used only in the beam–column load case. It is not applied horizontally to the wheelchair and does not enter the tipping equations. Wheelchair tipping is evaluated separately using the explicitly prescribed abrupt-stop, one-sided curb-drop, and uphill-slope scenarios in Section 3. The equivalent compressive load used in the beam–column model is then

(7) $$P_{\mathrm{eq}}=F_{t}+F_{d,v}.$$

The average axial compressive stress is

(8) $$\sigma_{c}=\frac{P_{\mathrm{eq}}}{A_{c}},$$

where $A_{c}$ is the effective cross-sectional area of the vertical support. The Euler critical load is

(9) $$P_{cr}=\frac{\pi^{2}EI_{\min}}{{\left(KL_{c}\right)}^{2}},$$

where *E* is Young’s modulus, $I_{\min}$ is the smaller principal second moment of area, $L_{c}$ is the unsupported vertical length, and *K* is the effective-length factor. For the fixed–free cantilever idealization adopted in this study, K=2.

The first-order moment caused by the horizontal offset of the applied load is

(10) $$M_{0}=P_{\mathrm{eq}}e_{\mathrm{top}},$$

where $e_{\mathrm{top}}$ is the horizontal distance from the centroidal axis of the vertical member to the upper load line. For $P_{\mathrm{eq}}<P_{\mathrm{cr}}$, a reduced-order elastic moment magnification is used to represent the *P*–Δ effect:

(11) $$M_{\mathrm{amp}}=\frac{M_{0}}{1-P_{\mathrm{eq}}/P_{\mathrm{cr}}}.$$

The corresponding extreme-fiber compressive stress is

(12) $$\sigma_{\max}=\frac{P_{\mathrm{eq}}}{A_{c}}+\frac{M_{\mathrm{amp}}c}{I_{\min}},$$

where *c* is the distance from the neutral axis to the extreme fiber. The Euler buckling and combined beam–column factors of safety are defined as

(13) $$FoS_{\mathrm{buckling}}=\frac{P_{\mathrm{cr}}}{P_{\mathrm{eq}}},$$

and

(14) $$FoS_{\mathrm{combined}}=\frac{\eta_{w}\sigma_{y}}{\sigma_{\max}},$$

respectively, where $\sigma_{y}$ is the material yield strength and $\eta_{w}$ is the fabrication-efficiency factor. The combined factor of safety accounts for direct compression, eccentric bending, and elastic moment magnification, whereas $FoS_{\mathrm{buckling}}$ provides a separate global Euler-instability screening metric.

Equation (6) provides a quasi-static representation of mobility-related inertial loading. It does not resolve the time-dependent response associated with wheel–floor impacts, caster rotation, patient motion, caregiver-applied forces, or structural vibration. The adopted vertical-acceleration range therefore serves as an engineering screening envelope for examining the sensitivity of the predicted beam–column response to mobility-related shock loading. It is distinct from the fixed conditional tipping scenarios.

## 3. Wheelchair Geometry and Scenario-Based Tipping Stability

Generic dimensions for a conventional 18 in adult hospital wheelchair were obtained from publicly available online specifications [25,26]. The reported dimensions indicate an overall length of approximately 42 in with the front rigging installed, an overall width of approximately 26–26.5 in, an overall height of approximately 35–36 in, a seat depth of 16–18 in, a seat-to-floor height of 17.5–19.5 in, 8 in front casters, and 24 in rear wheels. These dimensions were adopted as representative values for defining the wheelchair support geometry used in the scenario-based tipping-stability calculations. The dimensions used in the reduced-order model are summarized in Table 1.

The published overall length includes the leg rests and is not the longitudinal tipping wheelbase. Similarly, overall width is measured between the outermost points and is not the wheel-contact track. Because manufacturers do not report the contact dimensions for these hospital-chair models, conservative nominal values of $L_{f}=18$ in and B=24 in were adopted and bracketed by the ranges in Table 1. The 36 lb manufacturer value is the mass of the unmodified wheelchair; the model quantity $W_{s}=95$ lb represents the wheelchair together with the attached HALO support hardware and therefore must not be interpreted as the mass of the bare chair.

Let $W_{i}$, $x_{i}$, $y_{i}$, and $h_{i}$ denote the weight and center-of-gravity coordinates of component *i*, where i∈{p,s,w} represents the patient, wheelchair/HALO support system, and guided traction-weight assembly, respectively. The longitudinal coordinate origin is at the original rear-wheel axle and positive *x* is directed rearward. The traction force is internal to the complete chair–patient–HALO free body and, because its line is aligned above the patient, it is not introduced as a separate overturning moment.

Forward tipping was evaluated for an abrupt stop about the front-caster contact line, located at $x_{f}=-L_{f}$. Here, $M_{R}$ denotes the restoring moment opposing forward rotation, whereas $M_{O}$ denotes the overturning moment promoting forward rotation. The index *i* represents each mass component of the wheelchair–patient–HALO system; $W_{i}$, $x_{i}$, and $h_{i}$ are its weight, longitudinal center-of-gravity location, and center-of-gravity height above the ground, respectively. For a prescribed deceleration ratio $a_{\mathrm{stop}}/g$, the moments about the front-caster contact line and the corresponding factor of safety are

(15) $$\begin{array}{cc} \;\;\;\;\;\;\;\;\;M_{R,f} & =\sum_{i}W_{i}\max\left(x_{i}-x_{f},0\right), \end{array}$$

(16) $$\begin{array}{cc} \;\;\;\;\;\;\;\;\;\;\;\;\;\;M_{O,f} & =\sum_{i}W_{i}\max\left(x_{f}-x_{i},0\right)+\frac{a_{\mathrm{stop}}}{g}\sum_{i}W_{i}h_{i}, \end{array}$$

(17) $$\begin{array}{cc} FoS_{f} & =\frac{M_{R,f}}{M_{O,f}}. \end{array}$$

The first term in $M_{O,f}$ accounts for any gravitational load located forward of the front-caster tipping axis, whereas the second term represents the overturning moment produced by the effective inertial forces during deceleration. Conversely, the terms included in $M_{R,f}$ arise from weights whose centers of gravity remain behind the tipping axis and therefore oppose forward rotation.

A fixed value of $a_{\mathrm{stop}}=0.25g$ (approximately $2.45\;m/s^{2}$) was used as a deliberately conservative abrupt-stop scenario. This value is slightly greater than the upper experimental deceleration of $2.2\;m/s^{2}$ reported in a wheelchair fall-risk study [27]; it is therefore a conditional stability-screening condition and is not intended to represent routine attendant propulsion.

Lateral tipping was evaluated as a conditional misuse/obstacle scenario in which one side of the chair drops a height *d* while the opposite wheel remains on the upper surface. Wheelchair curb-descent experiments have considered drop heights of 0.05, 0.10, and 0.15 m [28,29]. The screening model uses d=2 in (approximately 0.05 m), giving

(18) $$\begin{array}{cc} \phi & =\sin^{-1}\;\left(\frac{d}{B}\right), \end{array}$$

(19) $$\begin{array}{cc} M_{R,\ell } & =\cos\phi \sum_{i}W_{i}\max\;\left(\frac{B}{2}-y_{i},0\right), \end{array}$$

(20) $$\begin{array}{cc} M_{O,\ell } & =\cos\phi \sum_{i}W_{i}\max\;\left(y_{i}-\frac{B}{2},0\right)+\sin\phi \sum_{i}W_{i}h_{i}, \end{array}$$

(21) $$\begin{array}{cc} FoS_{\ell } & =\frac{M_{R,\ell }}{M_{O,\ell }}. \end{array}$$

This quasi-static expression does not attempt to reproduce the short-duration impact acceleration when the lower wheel contacts the ground.

Rearward tipping was evaluated on a 10-degree uphill slope about the extended rear support at $x_{rc}$. The 10-degree condition is consistent with wheelchair stability test practice [30]. With $\theta =10^{\circ }$,

(22) $$\begin{array}{cc} M_{R,r} & =\cos\theta \sum_{i}W_{i}\max\left(x_{rc}-x_{i},0\right), \end{array}$$

(23) $$\begin{array}{cc} M_{O,r} & =\cos\theta \sum_{i}W_{i}\max\left(x_{i}-x_{rc},0\right)+\sin\theta \sum_{i}W_{i}h_{i}, \end{array}$$

(24) $$\begin{array}{cc} FoS_{r} & =\frac{M_{R,r}}{M_{O,r}}. \end{array}$$

The governing conditional tipping factor of safety is

(25) $$FoS_{\mathrm{tip}}=\min\left(FoS_{f},FoS_{\ell },FoS_{r}\right).$$

Table 2 gives deterministic checks at the representative geometry and mass distribution. The abrupt-stop case governs the nominal conditional envelope, but its factor of safety remains 4.67 at 0.25g. The rearward uphill case has a factor of safety of 5.61 and therefore does not govern. A deliberately extreme illustration in which a wheelchair traveling at 10 m/s is brought uniformly to rest in 1 s corresponds to $a_{\mathrm{stop}}/g=1.02$ and gives $FoS_{f}=1.14$. This falls below the adopted screening margin of 1.5 but remains above the physical moment-balance tipping threshold of unity; the nominal forward-tip threshold is approximately 1.17g. Thus, even this runaway illustration approaches forward, not rearward, instability.

Only the first three prescribed scenarios enter the reported conditional UQ envelope; the 6 in drop and runaway stop are deterministic stress-test illustrations.

These calculations are scenario-based stability screens. They do not assign occurrence probabilities to emergency braking, a one-sided curb drop, or slope exposure, and therefore should not be interpreted as accident probabilities. Accordingly, $FoS_{\mathrm{tip}}$ and its three component scenarios are reported separately and are not inserted into the primary probabilistic structural and pin-interface minimum,

(26) $$FoS_{\min,\mathrm{struct}}=\min\left(FoS_{\mathrm{buckling}},FoS_{\mathrm{combined}},FoS_{\mathrm{weld}},FoS_{\mathrm{pin}}\right).$$

For transparency, the code also returns the conditional scenario envelope

(27) $$FoS_{\min,\mathrm{with}\;\mathrm{tip}}=\min\left(FoS_{\min,\mathrm{struct}},FoS_{\mathrm{tip}}\right),$$

but this value is not used as the ordinary-use failure probability or in the governing structural-mode figure.

## 4. Welded-Joint and Fatigue Screening

The welded joints connecting the vertical support member, rear structural frame, rear-caster supports, and wheelchair-attachment members form important parts of the structural load path. During operation, these connections may be subjected to static loading from the prescribed traction force together with load variations caused by wheelchair motion, patient movement, and repeated loading and unloading. The present reduced-order model therefore includes a static weld-capacity limit state and a separate fatigue comparison metric.

The effective weld-throat area is approximated as

(28) $$A_{w}=0.707h_{\mathrm{weld}}L_{\mathrm{weld}},$$

where $h_{\mathrm{weld}}$ is the nominal leg size of an equal-leg fillet weld and $L_{\mathrm{weld}}$ is the effective weld length. The factor $0.707=1/\sqrt{2}$ follows from the geometry of a $45^{\circ }$ equal-leg fillet weld: the minimum effective throat thickness is $t_{\mathrm{throat}}=h_{\mathrm{weld}}\sin45^{\circ }=0.707h_{\mathrm{weld}}$. Thus, $A_{w}=t_{\mathrm{throat}}L_{\mathrm{weld}}$. This expression assumes an ideal equal-leg fillet-weld profile and does not account for local defects, incomplete fusion, or variations in the actual weld geometry.

The average weld-throat shear stress is then approximated by

(29) $$\tau_{w}=\frac{P_{\mathrm{eq}}}{A_{w}},$$

where $P_{\mathrm{eq}}$ is the equivalent load defined in Equation (7). This formulation represents a simplified average-stress calculation and does not resolve the local weld geometry, stress concentration, residual stress, or multiaxial stress state.

The weld factor of safety is defined as

(30) $$FoS_{\mathrm{weld}}=\frac{\eta_{w}\tau_{\mathrm{allow}}}{\tau_{w}},$$

where $\tau_{\mathrm{allow}}$ is the nominal allowable weld shear stress and $\eta_{w}$ is a fabrication-efficiency factor. The corresponding weld limit-state function is

(31) $$g_{\mathrm{weld}}=\eta_{w}\tau_{\mathrm{allow}}-\tau_{w}.$$

A realization satisfying $g_{\mathrm{weld}}<0$, equivalently $FoS_{\mathrm{weld}}<1$, is classified as a weld-limit-state violation. The weld factor of safety is included in the governing minimum factor of safety and the binary system failure indicator.

Repeated wheelchair use may also produce cyclic stresses. However, the available loading information is insufficient to define a validated stress spectrum or fatigue-life model for the prototype. The reduced-order model therefore calculates only a fatigue comparison metric. A representative alternating stress is approximated as

(32) $$\sigma_{\mathrm{alt}}=f_{\mathrm{fat}}\sigma_{\mathrm{lat}},$$

where $f_{\mathrm{fat}}$ is a prescribed stress fraction and $\sigma_{\mathrm{lat}}$ is the reduced-order lateral bending stress. The corresponding fatigue comparison factor is

(33) $$FoS_{\mathrm{fatigue}}=\frac{\sigma_{\mathrm{endurance}}}{\sigma_{\mathrm{alt}}},$$

where $\sigma_{\mathrm{endurance}}$ is the assumed material endurance strength.

The fatigue comparison is retained as a diagnostic output but is not included as a governing failure mode in $FoS_{\min,\mathrm{struct}}$ or $P_{f}$. The model does not calculate fatigue life, cumulative damage, weld-detail category effects, or cycle-dependent crack growth. A defensible fatigue-life assessment would require measured operational load histories, local weld stresses, appropriate weld fatigue classifications, and a cumulative-damage model. Accordingly, the present weld and fatigue calculations should be interpreted as screening-level engineering metrics rather than as validation of welded-joint life.

## 5. Coupled Clinical–Structural Uncertainty Model

The prototype halo-gravity traction (HGT) wheelchair is modeled as a coupled clinical–structural system rather than solely as a static mechanical structure. Its engineering response involves prescribed traction delivery, combined structural loading, buckling resistance, welded-joint capacity, scenario-based wheelchair tipping stability, and halo-pin load transfer. These responses depend on patient-related loading parameters, device geometry, fabrication-related properties, and mobility-related operating conditions. The present framework therefore evaluates how prescribed uncertainties in these inputs propagate to the principal structural and clinical surrogate response measures.

The uncertainty framework considers three broad categories of input uncertainty:

1. Patient-related and clinically informed loading uncertainty;
2. Device geometry and fabrication-related uncertainty; and
3. Operational and mobility-related uncertainty.

Patient-related inputs include body weight, prescribed traction ratio, effective spinal stiffness, and the number of active halo pins. Device-related inputs include effective structural-section properties, load eccentricities, rear-support and weight-stack locations, wheelchair wheelbase and track, weld/fabrication efficiency, and halo-pin load-sharing parameters. Operational uncertainty is represented through adverse vertical acceleration of the guided weight carriage. The abrupt-stop deceleration, one-sided curb-drop height, and uphill slope are fixed conditional tipping scenarios rather than random ordinary-use events.

Unlike a conventional structural analysis based exclusively on nominal material and geometric values, the present framework propagates these clinically informed, structural, and operational uncertainties simultaneously through the reduced-order model. This coupled formulation provides a broader engineering assessment of the interactions among traction delivery, beam–column response, buckling, weld loading, tipping stability, and halo-pin load concentration. The resulting quantities are conditional screening-level predictions and should not be interpreted as experimentally validated measures of clinical safety or operational reliability.

### 5.1. Clinical Input Variables

The patient-related uncertain input vector is defined as

(34) $$X_{c}=\left\{W_{p},\;r_{t},\;K_{s},\;N_{p}\right\},$$

where $W_{p}$ denotes patient body weight, $r_{t}$ is the prescribed traction-to-body-weight ratio, $K_{s}$ is an effective spinal-stiffness surrogate, and $N_{p}$ is the number of active halo pins. The prescribed ranges of these quantities are reported in Table 3.

The prescribed traction force is calculated deterministically from patient weight and traction ratio as

(35) $$F_{t}=r_{t}W_{p},$$

with

(36) $$0.20\leq r_{t}\leq 0.50.$$

The uncertain inputs $W_{p}$ and $r_{t}$ are sampled independently in the present uncertainty model. Once sampled, however, the traction force $F_{t}$ is calculated from Equation (35) and is therefore not treated as an additional independent random variable. This deterministic relationship preserves the direct dependence of the prescribed traction force on patient weight and the selected traction ratio.

The number of active pins is treated as a discrete uncertain quantity. A latent beta-distributed sample is first mapped to the interval $4\leq N_{p}\leq 8$ and then rounded to the nearest integer before model evaluation. The resulting realized values of $N_{p}$ are therefore discrete, even though the underlying latent sample is generated using the same bounded beta-distribution procedure as the continuous inputs.

Only clinical variables that directly enter the reported response and limit-state calculations were included in the uncertainty-propagation model.

### 5.2. Clinical Response Approximation

To provide a representative measure of the coupling between prescribed traction and an idealized clinical response, a spinal-displacement surrogate is defined as

(37) $$\Delta L_{s}=\frac{F_{t}}{K_{s}},$$

where $F_{t}$ is the prescribed traction force and $K_{s}$ is an effective linear spinal-stiffness surrogate. The normalized clinical-response metric is then defined as

(38) $$\eta_{c}=\frac{\Delta L_{s}}{\Delta L_{\mathrm{target}}},$$

where the fixed normalization value used in the present model is

(39) $$\Delta L_{\mathrm{target}}=1.5\;\mathrm{in}.$$

The quantities $\Delta L_{s}$ and $\eta_{c}$ are reduced-order surrogate responses introduced to illustrate how uncertainty in patient weight, traction ratio, and effective spinal stiffness propagates through the coupled framework. They do not represent a validated biomechanical model of spinal deformation or correction. In particular, the linear relationship does not account for patient-specific anatomy, nonlinear tissue response, deformity geometry, viscoelastic effects, treatment duration, growth, or time-dependent clinical adaptation. Accordingly, $\eta_{c}$ should not be interpreted as a predicted percentage of clinical correction or as a patient-specific treatment outcome.

### 5.3. Device, Manufacturing, and Operational Variables

The device-, fabrication-, and operation-related uncertain input vector is defined as

(40) $$X_{d}=\left\{F_{\mathrm{pin},\mathrm{eng}},\;e_{\mathrm{pin}},\;\eta_{\mathrm{pin}},\;A_{c},\;I_{\min},\;e_{\mathrm{top}},\;\eta_{w},\;x_{rc},\;x_{w,\mathrm{cg}},\;a_{g,v},\;L_{f},\;B\right\}.$$

Together with the four patient-related inputs in Equation (34), this vector gives a total of 16 uncertain model inputs.

The parameter $F_{\mathrm{pin},\mathrm{eng}}$ is the engineering pin–skull interface screening load, $e_{\mathrm{pin}}$ is the pin-load amplification factor, and $\eta_{\mathrm{pin}}$ is the halo-pin load-sharing efficiency. These quantities represent uncertainty in the reduced-order halo-pin load-transfer model. The parameter $F_{\mathrm{pin},\mathrm{eng}}$ is not interpreted as a clinically validated allowable load for an individual pediatric skull pin.

The variables $A_{c}$ and $I_{\min}$ denote the effective cross-sectional area and weak-axis second moment of area of the vertical support member, respectively. The parameter $e_{\mathrm{top}}$ is the horizontal offset between the centroidal axis of the vertical support and the upper load line, while $\eta_{w}$ is the weld/fabrication efficiency factor.

The variables $x_{rc}$, $x_{w,\mathrm{cg}}$, $L_{f}$, and *B* describe the wheelchair support and load geometry: the rear anti-tip support location, guided weight-stack center-of-gravity location, rear-axle-to-front-caster wheelbase, and rear-wheel contact track, respectively. The track *B* is the transverse center-to-center distance between the left and right rear-wheel ground-contact regions. A nominal value of B=24 in was used, with a range of 23–25 in in the uncertainty analysis. Consequently, B/2 is the lateral distance from the wheelchair centerplane to either rear-wheel contact line used in the lateral-tipping calculation.

The operational parameter $a_{g,v}=a_{v}/g$ denotes the adverse vertical acceleration of the guided weight carriage normalized by gravitational acceleration and enters only the beam–column compression envelope. The horizontal stop deceleration is fixed at 0.25g in the forward-tipping scenario and is not sampled as $a_{g,v}$. The traction force is internal to the complete wheelchair–patient–HALO free body; therefore, no separate traction-line eccentricity is included in the tipping moment balance.

The effective area $A_{c}$ and weak-axis second moment $I_{\min}$ were sampled independently as reduced-order section properties. Because both quantities describe the same structural member, independent sampling may admit combinations that do not correspond exactly to a single realizable C-channel geometry. This treatment is retained as a screening-level approximation and is identified as a limitation of the present model. A higher-fidelity implementation would sample physical section dimensions and calculate $A_{c}$ and $I_{\min}$ deterministically.

The elastic modulus, unsupported column length, and fixed–free effective-length factor are treated as deterministic quantities, with nominal values

(41) $$E=29\;\mathrm{Msi},\;L_{c}=68\;\mathrm{in}.,\;K=2.$$

These quantities were held fixed to limit the dimensionality of the present screening analysis and because experimentally supported distributions were not available for the prototype. Their exclusion from the uncertainty vector is a modeling assumption rather than evidence that their variability is negligible.

The relationship between prescribed traction force and guided weight-stack load is represented as

(42) $$F_{t}=\eta_{p}F_{w},$$

or equivalently,

(43) $$F_{w}=\frac{F_{t}}{\eta_{p}}.$$

The present implementation uses the fixed nominal pulley efficiency

(44) $$\eta_{p}=0.95.$$

Pulley efficiency is therefore not propagated as an uncertain input in the reported analysis. Equation (42) is used only to determine the weight-stack load transmitted through the internal structural load path. The reported delivered traction force remains equal to the prescribed value $F_{t}=r_{t}W_{p}$.

### 5.4. Halo-Pin Load Model

The halo-pin subsystem provides the mechanical interface through which the prescribed traction force is transmitted to the patient. Because the available traction load is distributed among a finite number of fixation pins, uncertainty in the number of active pins and the degree of load sharing can produce substantial variation in the estimated load carried by an individual pin. The present formulation is intended only as a reduced-order engineering screening model of traction-induced pin loading.

Under the idealized assumption of equal load sharing among $N_{p}$ active pins, the average traction-induced load per pin is

(45) $$F_{\mathrm{pin},\mathrm{avg}}=\frac{F_{t}}{N_{p}}.$$

In practice, geometric misalignment, differences in pin position and insertion, deformation of the halo assembly, and local anatomical variation may produce unequal load sharing. The estimated maximum traction-induced load carried by an individual pin is therefore represented by

(46) $$F_{\mathrm{pin},\max}=e_{\mathrm{pin}}\frac{F_{t}}{\eta_{\mathrm{pin}}N_{p}},$$

where $e_{\mathrm{pin}}$ is a pin-load amplification factor and $\eta_{\mathrm{pin}}$ is the pin load-sharing efficiency. The idealized equal-sharing condition corresponds to $e_{\mathrm{pin}}=1$ and $\eta_{\mathrm{pin}}=1$. Increasing $e_{\mathrm{pin}}$ or decreasing $\eta_{\mathrm{pin}}$ produces a larger estimated maximum pin load.

The halo-pin engineering screening factor is defined as

(47) $$FoS_{\mathrm{pin}}=\frac{F_{\mathrm{pin},\mathrm{eng}}}{F_{\mathrm{pin},\max}},$$

where $F_{\mathrm{pin},\mathrm{eng}}$ is the uncertain engineering pin–skull interface screening load. The corresponding limit-state function is

(48) $$g_{\mathrm{pin}}=F_{\mathrm{pin},\mathrm{eng}}-F_{\mathrm{pin},\max}.$$

A realization satisfying $g_{\mathrm{pin}}<0$, equivalently $FoS_{\mathrm{pin}}<1$, is classified as a pin-model limit-state violation within the computational screening framework.

The quantities $F_{\mathrm{pin},\mathrm{avg}}$ and $F_{\mathrm{pin},\max}$ represent traction-induced load measures and should not be interpreted as the axial pin preload generated during halo installation. Moreover, $F_{\mathrm{pin},\mathrm{eng}}$ is not a clinically validated allowable load or a universal pediatric safety threshold. The model does not resolve skull thickness, bone quality, pin-tip geometry, insertion angle, insertion torque, local contact stress, time-dependent loosening, or combined axial and transverse loading. Consequently, $FoS_{\mathrm{pin}}$ is interpreted only as an engineering screening metric for the simplified halo-pin load-transfer model.

#### Interpretation of Halo-Pin Loading and Engineering Load Limits

The mechanical loading of a halo pin should be distinguished from the total traction load applied to the patient. Insertion torque generates an approximately radial axial force that acts as a compressive preload at the pin–skull interface. This preload is distinct from the vertical traction-induced load subsequently transmitted through the halo assembly; therefore, insertion torque, axial pin force, and vertical traction load should not be treated as equivalent quantities.

Karnes et al. [31] investigated the relationship between initial halo-pin force and the vertical force required to disengage a four-pin halo from cadaver heads. Initial axial pin forces of 120, 240, and 360 N (approximately 27, 54, and 81 lbf per pin) produced predicted disengagement forces of approximately 80, 320, and 570 N (18, 72, and 128 lbf), respectively. Based on the lower 95% prediction interval and a previously reported maximum vertical halo load of 186 N (41.8 lbf), the authors estimated that an average initial pin force of approximately 230 N (51.7 lbf) was required to prevent disengagement.

Semmelink et al. [32] reported that an axial tightening force of 100 N (22.5 lbf), corresponding to approximately 0.5 in-lb insertion torque, required at least 200 N (45 lbf) of vertical traction to produce pin migration. An axial tightening force of 200 N (45 lbf), corresponding to approximately 2 in-lb, resisted the maximum applied vertical traction force of 360 N (81 lbf) for all but one tested pin. They also associated an insertion torque of approximately 4 in-lb with an axial pin force of approximately 350 N (79 lbf). Their axial load-to-failure tests produced failure loads of approximately 780–1270 N (175–285 lbf) in adult cadaver specimens. These results establish the approximate scale of mechanical interface failure under those experimental conditions. Based on the available evidence, the present engineering screening interval was set to 50–175 lbf.

For illustration, an 80 lb patient subjected to traction equal to 50% of body weight through eight pins would have an idealized average vertical load of

(49) $$F_{v,\mathrm{pin}}=\frac{0.50(80)}{8}=5\;\mathrm{lbf}/\mathrm{pin}.$$

This load is fundamentally different from the radial axial preload generated during installation. Because the cited experiments used adult cadaver skulls and primarily characterized preload, migration, disengagement, or axial failure under specific test conditions, this comparison is only a dimensional screening surrogate. It is not a validated interaction criterion for combined preload and traction-induced loading or a clinically validated pediatric safety threshold.

### 5.5. Combined System Response

The reduced-order framework combines the patient-related and device-level models through the input–output mapping

(50) $$Y=f\;\left(X_{c},\;X_{d}\right),$$

where $X_{c}$ and $X_{d}$ denote the patient-related and device-, fabrication-, and operation-related uncertainty vectors, respectively. This mapping propagates the prescribed input uncertainties to the structural screening measures, traction-delivery quantities, and clinical surrogate responses.

The principal response vector is defined as

(51) $$\begin{array}{cc} Y=\{ & P_{\mathrm{eq}},\;\sigma_{c},\;\sigma_{\max},\;P_{\mathrm{cr}},\;FoS_{\mathrm{buckling}},\;FoS_{\mathrm{combined}},\;\\ \; & FoS_{\mathrm{weld}},\;FoS_{\mathrm{pin}},\;FoS_{\min,\mathrm{struct}},\;\\ \; & FoS_{f},\;FoS_{\ell },\;FoS_{r},\;FoS_{\mathrm{tip}},\;F_{t,d},\;\Delta L_{s},\;\eta_{c},\;I_{f}\}. \end{array}$$

Here, $F_{t,d}$ denotes the reported delivered traction force, $\Delta L_{s}$ and $\eta_{c}$ are clinical surrogate responses, and $I_{f}$ is a binary indicator of whether any primary structural or pin-interface limit state is violated. The four tipping quantities are conditional scenario outputs and are not included in this ordinary-use indicator.

The governing minimum factor of safety is calculated for each realization as

(52) $$FoS_{\min,\mathrm{struct}}=\min\left\{FoS_{\mathrm{buckling}},\;FoS_{\mathrm{combined}},\;FoS_{\mathrm{weld}},\;FoS_{\mathrm{pin}}\right\}.$$

The required buckling factor of safety is 2.0, whereas the combined-stress, weld, and halo-pin screening criteria use a threshold of unity. The conditional tipping envelope is evaluated separately against $FoS_{\mathrm{tip}}=1.5$ for each prescribed scenario.

The governing engineering limit-state functions are

(53) $$G_{\mathrm{struct}}=\left\{g_{\mathrm{buckling}},\;g_{\mathrm{combined}},\;g_{\mathrm{weld}},\;g_{\mathrm{pin}}\right\}.$$

For a realization X, the system-level violation indicator is defined as

(54) $$I_{f}\left(X\right)=\left\{\begin{array}{cc} 1,\; & \mathrm{if}\;\mathrm{at}\;\mathrm{least}\;\mathrm{one}\;g_{j}\left(X\right)<0,\;g_{j}\in G_{\mathrm{struct}},\\ 0,\; & \mathrm{otherwise}. \end{array}\right.$$

Equivalently,

(55) $$I_{f}\left(X\right)=\max_{g_{j}\in G_{\mathrm{struct}}}I\;\left[g_{j}\left(X\right)<0\right],$$

where I[·] is the indicator function. For beta-shape case *k*, the conditional model-based probability of a limit-state violation is estimated from *N* realizations as

(56) $${\hat{P}}_{f,k}=\frac{1}{N}\sum_{n=1}^{N}I_{f}\;\left(X_{k}^{\left(n\right)}\right).$$

Thus, the quantity stored by the computational model for each realization is the binary indicator $I_{f}$, while its sample mean provides the estimated conditional probability ${\hat{P}}_{f,k}$. Because the input bounds and beta-distribution shapes are prescribed engineering uncertainty models, ${\hat{P}}_{f,k}$ should not be interpreted as an empirically established population failure probability.

### 5.6. Modeling Scope

The proposed formulation is a reduced-order engineering uncertainty-screening framework for the halo-gravity traction wheelchair. It is intended to examine how prescribed patient-related, operational, geometric, fabrication, and halo-interface uncertainties propagate through simplified representations of the principal load paths and engineering limit states. The framework is not intended to predict patient-specific clinical outcomes, establish population-level failure probabilities, or certify the safety of the prototype.

The beam–column, buckling, weld, tipping, and halo-pin formulations retain the principal mechanical relationships required for uncertainty propagation and global sensitivity analysis while avoiding the computational cost of a full-system finite-element or multibody model. The tipping calculation is a quasi-static support-polygon model and does not resolve transient wheel–ground interaction or impact dynamics. The halo-pin calculation is an engineering load-transfer surrogate and does not represent a clinically validated pediatric pin–skull failure criterion. Similarly, the clinical response metric is an idealized linear-stiffness surrogate rather than a patient-specific biomechanical prediction.

Accordingly, the reported factors of safety, limit-state indicators, response distributions, and sensitivity indices are conditional on the adopted reduced-order equations, input bounds, beta-distribution shapes, and independence assumptions. The framework is intended to support preliminary engineering evaluation, identify influential parameters and competing response modes, and guide subsequent model refinement, higher-fidelity simulation, prototype load testing, and dynamic stability testing.

### 5.7. Design Implications

The principal methodological contribution of the present work is the development of a unified reduced-order framework for propagating patient-related, operational, geometric, fabrication, and halo-interface uncertainties through interacting engineering response models for a mobile halo-gravity traction system. The framework allows the relative influence of the uncertain inputs on beam–column response, buckling, weld loading, scenario-based tipping, halo-pin load concentration, traction delivery, and the clinical surrogate response to be examined within a common computational structure.

The resulting response distributions and sensitivity indices provide screening-level guidance for subsequent design refinement. Parameters that strongly influence a particular response can be prioritized for improved measurement, tighter fabrication control, geometric redesign, operational restriction, or higher-fidelity analysis. In particular, the framework can help distinguish whether a predicted response is governed primarily by traction loading, structural-section properties, support-polygon geometry, mobility-related acceleration, fabrication efficiency, or assumptions concerning halo-pin load sharing.

The analysis does not replace deterministic design checks, applicable standards, prototype testing, or clinical evaluation. Rather, it complements nominal calculations by showing how the predicted engineering responses vary over the prescribed uncertainty space and across alternative beta-distribution shapes. The results can therefore be used to prioritize future finite-element modeling, static proof testing, dynamic tipping tests, weld inspection and fatigue characterization, halo-interface investigation, and refinement of the clinical surrogate model before any conclusions regarding device safety or clinical use are made.

## 6. Uncertainty Quantification Methodology

The halo-gravity traction wheelchair model contains prescribed uncertainty associated with patient-related loading, structural geometry, fabrication quality, halo-pin load transfer, support-polygon geometry, and mobility-related operating conditions. A deterministic calculation based only on nominal inputs would not characterize the variation in the predicted engineering responses over these prescribed ranges. Uncertainty quantification is therefore used to propagate the input variability through the reduced-order model and evaluate its influence on the principal structural screening measures and clinical surrogate responses.

The complete uncertain input vector is

(57) $$\begin{array}{cc} X=\{ & W_{p},\;r_{t},\;K_{s},\;N_{p},\;F_{\mathrm{pin},\mathrm{eng}},\;e_{\mathrm{pin}},\;\eta_{\mathrm{pin}},\;A_{c},\;I_{\min},\;\\ \; & e_{\mathrm{top}},\;\eta_{w},\;x_{rc},\;x_{w,\mathrm{cg}},\;a_{g,v},\;L_{f},\;B\}. \end{array}$$

The physical interpretation and bounds of the 16 uncertain inputs are summarized in Table 3.

### 6.1. Basis and Interpretation of the Uncertain Inputs

The intervals in Table 3 combine literature-informed clinical and biomechanical ranges, prototype-specific geometric bounds, and bounded engineering assumptions. They should not all be interpreted as statistically measured population distributions. The beta distributions provide bounded sampling models over the prescribed intervals; where population-level data are unavailable, the intervals represent epistemic uncertainty in the reduced-order model.

Current pediatric HGT guidance recommends beginning with small traction loads and progressively increasing the load toward approximately 50% of patient body weight [33]. Shaw et al. likewise identify 50% of body weight as a maximum applied traction level in pediatric spinal deformity [34]. Han et al. reported a mean traction weight of 39.9±11.1% of body weight [35], and Popescu et al. increased traction to 40–50% of body weight [36]. Thus, $r_{t}=0.20$–0.50 spans a progressive-loading range from moderate traction to the commonly reported clinical target. The patient-weight interval $W_{p}=50$–100 lb is a design-population bound for the mobility system, not a statistical characterization of all pediatric HGT patients.

The number of active pins is also literature-informed. Pediatric guidance recommends at least six pins in skeletally immature children and considers eight or more pins in younger children, with pin number and insertion torque adjusted for age and bone quality [33,34]. Four-pin configurations nevertheless appear in clinical and experimental reports [36,37]. The interval $N_{p}=4$–8 was retained to span configurations relevant to this engineering analysis rather than to prescribe a preferred clinical configuration.

The engineering pin–skull screening-load interval is supported by the mechanical evidence discussed in Section 5.4. Karnes et al. estimated that an initial pin force of approximately 230 N (51.7 lbf) was required to resist previously reported vertical halo loading [31]. Semmelink et al. related a 4 in-lb insertion torque to an axial pin force of approximately 350 N (79 lbf) and reported axial pin–skull failure loads of approximately 780–1270 N (175–285 lbf) [32]. Accordingly, $F_{\mathrm{pin},\mathrm{eng}}=50$–175 lbf extends from experimentally reported fixation-force levels to approximately the lower end of the reported adult cadaver failure range. It remains an engineering screening interval, not a clinically validated allowable load for an individual pediatric halo pin.

Population distributions are unavailable for the pin-load amplification and load-sharing parameters. Kuester et al. demonstrated that the halo is an over-constrained mechanical system in which individual pin forces evolve differently under transverse loading [37]. Therefore, $e_{\mathrm{pin}}=1$–3 and $\eta_{\mathrm{pin}}=0.50$–1.00 are treated as bounded epistemic factors representing departures from ideal concentric, equal load sharing.

The effective spinal stiffness $K_{s}$ is a reduced-order surrogate rather than a directly measurable clinical stiffness. Axial-suspension testing has shown substantial inter-patient variability in the mechanical response of scoliotic spines [38]. Patient-specific finite-element studies and intraoperative load–displacement measurements likewise demonstrate variability in three-dimensional spinal stiffness and between spinal regions [39,40]. Because those measurements cannot be converted directly to the linear stiffness used here without additional geometric assumptions, $K_{s}=15$–45 lb/in is retained as a bounded effective-stiffness surrogate, not a fitted population distribution.

The geometry variables $A_{c}$, $I_{\min}$, $e_{\mathrm{top}}$, $x_{rc}$, $x_{w,\mathrm{cg}}$, $L_{f}$, and *B* describe the design and configuration envelope of the prototype. They are design-space uncertainties rather than clinical population variables. The effective area and weak-axis second moment were sampled independently, which may admit combinations that do not correspond exactly to one realizable cross section. A higher-fidelity implementation should sample physical section dimensions and calculate $A_{c}$ and $I_{\min}$ deterministically.

The fabrication factor $\eta_{w}=0.70$–1.00 represents bounded uncertainty in fabrication quality and departure from ideal nominal behavior. Welding standards recognize the roles of qualified procedures, fabrication, and inspection in welded-joint performance [41]. Without prototype-specific inspection or destructive-test statistics, the interval is treated as epistemic engineering uncertainty.

Finally, $a_{g,v}$ represents adverse vertical inertial loading of the guided weight carriage. Curb-descent tests have produced mean peak vertical head accelerations of approximately 1.3–1.7g [29]; wheelchair travel over common surfaces and obstacles also produces sustained vibration and transient shocks [42]. These measurements motivate a mobility-related acceleration envelope but do not measure the acceleration transmitted to this guided weight carriage. The adopted $a_{g,v}=0$–1.0 interval is therefore an engineering operational bound rather than a fitted distribution for the beam–column load envelope. It is not used as a horizontal wheelchair acceleration in the tipping calculations. The separate forward-tipping case uses a fixed 0.25g stop, while the lateral and rearward checks use fixed 2 in drop and 10-degree uphill scenarios, respectively. Larger short-duration impacts remain outside the quasi-static screening model.

#### Independence of the Uncertain Inputs

The uncertain inputs were sampled independently because joint probability distributions and experimentally established correlations are unavailable for the clinical, geometric, fabrication, and operational variables considered. Independence is therefore a modeling assumption, not an experimentally established property. Potential dependencies remain plausible: patient-level quantities may be clinically related, pin number may influence load sharing, and geometry variables derived from a common structural configuration may be correlated. Explicit deterministic relationships, such as $F_{t}=r_{t}W_{p}$, are retained in the governing equations rather than represented through additional random variables. The reported results are conditional on both the specified marginal uncertainty models and the independence assumption.

The adopted intervals combine clinically informed ranges, prototype-based geometric ranges, published mechanical evidence, and engineering assumptions. They are not uniformly derived from statistically measured population data. Accordingly, the input distributions represent bounded uncertainty models over prescribed intervals rather than fitted population distributions. The resulting response distributions and limit-state estimates are conditional on these bounds and distributional assumptions.

For each sampled realization, the prescribed traction force is calculated as

(58) $$F_{t}=r_{t}W_{p}.$$

The inputs $W_{p}$ and $r_{t}$ are sampled independently. The traction force $F_{t}$ is then calculated deterministically from their product and is not treated as an additional independent random variable.

Within the present reduced-order framework, the reported delivered traction force is assumed to equal the prescribed traction force:

(59) $$F_{t,d}=F_{t}.$$

Pulley efficiency is not introduced as a reduction in the reported delivered traction. Instead, the fixed pulley efficiency $\eta_{p}=0.95$ is used to calculate the guided weight-stack load:

(60) $$F_{w}=\frac{F_{t}}{\eta_{p}}.$$

The mobility-related inertial force associated with acceleration or deceleration of the guided weight carriage is represented by

(61) $$F_{d,v}=F_{w}a_{g,v},$$

where $a_{g,v}=a_{v}/g$ is vertical acceleration normalized by gravitational acceleration. This quasi-static term represents the first-order influence of mobility-related vertical acceleration on the beam–column calculation. It does not constitute a transient dynamic model of starts, stops, turns, floor transitions, curb impacts, or patient motion.

The principal uncertain response vector is defined as

(62) $$\begin{array}{cc} Y=\{ & FoS_{\mathrm{buckling}},\;FoS_{\mathrm{combined}},\;FoS_{\mathrm{weld}},\;FoS_{\mathrm{pin}},\;FoS_{\min,\mathrm{struct}},\;\\ \; & FoS_{f},\;FoS_{\ell },\;FoS_{r},\;FoS_{\mathrm{tip}},\;\\ \; & F_{t,d},\;F_{\mathrm{pin},\max},\;\Delta L_{s},\;\eta_{c},\;I_{f}\}. \end{array}$$

These responses include the principal structural screening factors, the four conditional tipping outputs, traction-delivery quantity, maximum traction-induced pin load, clinical surrogate quantities, and the binary structural/interface limit-state indicator.

For each realization, the descriptive minimum factor of safety is

(63) $$FoS_{\min,\mathrm{struct}}=\min\left\{FoS_{\mathrm{buckling}},\;FoS_{\mathrm{combined}},\;FoS_{\mathrm{weld}},\;FoS_{\mathrm{pin}}\right\}.$$

This expression is the primary structural and pin-interface minimum. The adopted criteria are

(64) $$\begin{array}{cccc} FoS_{\mathrm{buckling}} & <2.0,\; & FoS_{\mathrm{combined}} & <1.0,\\ FoS_{\mathrm{weld}} & <1.0,\; & FoS_{\mathrm{pin}} & <1.0. \end{array}$$

The binary indicator $I_{f}$ is assigned a value of unity if any of these four structural or pin-interface criteria is violated and zero otherwise. The sample mean of $I_{f}$ within each beta-shape case provides the corresponding conditional model-based estimate of limit-state-violation probability. These estimates should not be interpreted as empirically established clinical or population-level failure probabilities. Separately, $FoS_{\mathrm{tip}}<1.5$ denotes an inadequate margin under at least one prescribed tipping scenario; it is not included in $I_{f}$ because the model does not assign occurrence probabilities to the stop, drop, or slope events.

The response distributions are used to calculate descriptive statistics, compare competing response modes, and determine the relative influence of the uncertain inputs through Polynomial Chaos Expansion and Sobol sensitivity analysis. Because the underlying equations remain reduced-order screening models, the resulting sensitivity rankings identify influential model inputs rather than experimentally validated causal determinants of device safety.

### 6.2. Latin Hypercube Sampling and Beta Distributions

The uncertain inputs were sampled using Latin Hypercube Sampling (LHS) in conjunction with bounded beta distributions. LHS stratifies the unit interval for each input and independently permutes the resulting strata across the multidimensional sample. This procedure improves coverage of the prescribed input space relative to unstratified random sampling for a given number of realizations.

For each beta-distribution shape case, N=4000 LHS realizations were generated for the 16 uncertain inputs. A fixed random-number seed, rng(1), was used to make the sampling procedure reproducible. The uncertain inputs were sampled independently; therefore, no statistical correlation structure was imposed among the input variables.

For an input $X_{i}$ bounded by $x_{i}^{-}$ and $x_{i}^{+}$, an initial stratified sample $U_{i}\in \left[0,1\right]$ was transformed using the inverse cumulative distribution function of a beta random variable:

(65) $$\xi_{i}=F_{\mathrm{Beta}}^{-1}\left(U_{i};\alpha_{k},\beta_{k}\right),\;0\leq \xi_{i}\leq 1,$$

where $\alpha_{k}$ and $\beta_{k}$ are the beta-shape parameters for distributional-shape case *k*. The physical input was then obtained from

(66) $$X_{i}=x_{i}^{-}+\xi_{i}\left(x_{i}^{+}-x_{i}^{-}\right).$$

The beta probability-density function on the normalized interval is

(67) $$p\left(\xi ;\alpha ,\beta \right)=\frac{\xi^{\alpha -1}{\left(1-\xi \right)}^{\beta -1}}{B(\alpha ,\beta )},\;0\leq \xi \leq 1,$$

where B(α,β) is the beta function. Depending on the values of α and β, the bounded distribution can represent symmetric, skewed, uniform, boundary-concentrated, or centrally concentrated sampling over the prescribed interval.

The 32 beta-shape pairs examined in the analysis are listed in Table 4. Within each case *k*, the same pair $\left(\alpha_{k},\beta_{k}\right)$ was assigned to all 16 uncertain inputs. Thus, the analysis represents a common-shape distributional sensitivity study rather than a collection of independently fitted marginal distributions for the individual variables.

For $N_{p}$, the latent beta-distributed sample was mapped continuously to the interval [4,8] and then rounded to the nearest integer before model evaluation. Consequently, the realized pin-number distribution was discrete, even though its latent sampling variable was generated using the same bounded-beta procedure.

The 32 cases were treated as alternative uncertainty-model specifications. Response statistics, Polynomial Chaos Expansions, Sobol indices, and limit-state-violation estimates were calculated separately for each case. Where an across-case descriptive summary was reported, the valid realizations from all cases were concatenated with equal case weighting. Such aggregate quantities represent descriptive distributional-robustness summaries and should not be interpreted as results from an empirically established mixture distribution or as population-level probabilities.

### 6.3. Polynomial Chaos Expansion

A non-intrusive Polynomial Chaos Expansion (PCE) was fitted separately for each scalar model response and each of the 32 beta-distribution shape cases. The PCE provides a spectral surrogate of the reduced-order model response and permits response moments and variance-based sensitivity indices to be calculated directly from the expansion coefficients.

For a scalar response *Y*, the PCE is written as

(68) $$Y\left(\xi \right)\approx \hat{Y}\left(\xi \right)=\sum_{\nu \in A_{p}}c_{\nu }\Psi_{\nu }\left(\xi \right),$$

where ξ is the vector of normalized uncertain inputs, $c_{\nu }$ is the coefficient associated with multi-index ν, and $\Psi_{\nu }\left(\xi \right)$ is the corresponding multivariate orthonormal polynomial basis function.

A total-order basis of polynomial order p=3 was used:

(69) $$A_{p}=\left\{\nu \in N_{0}^{16}:{∥\nu ∥}_{1}\leq 3\right\}.$$

For d=16 uncertain inputs, the number of retained basis terms was

(70) Nbasis=d+pp=16+33=969.

Each PCE was fitted using the N=4000 valid LHS model evaluations available for the corresponding response and beta-shape case. The regression problem was therefore overdetermined, with approximately 4.1 model evaluations per basis coefficient.

Because the normalized uncertain inputs follow beta distributions, Jacobi polynomials were used as the univariate basis functions. The physical samples were first mapped from their bounded physical intervals to [−1,1]. For a beta distribution with shape parameters $\left(\alpha_{k},\beta_{k}\right)$ on [0,1], the corresponding Jacobi weight on [−1,1] is proportional to

(71) $${\left(1-\xi \right)}^{\beta_{k}-1}{\left(1+\xi \right)}^{\alpha_{k}-1}.$$

The multivariate basis functions were formed as tensor products of the univariate Jacobi polynomials and normalized with respect to the target probability measure.

The expansion coefficients were estimated by ordinary least squares using a reduced QR factorization of the orthonormal regression matrix:

(72) $$A=QR,\;\hat{c}=R^{-1}Q^{T}y,$$

where A is the PCE design matrix, y contains the direct reduced-order model evaluations, and $\hat{c}$ is the fitted coefficient vector.

Because the basis is orthonormal, the PCE mean and variance are obtained from

(73) $${\hat{\mu }}_{Y}=c_{0},$$

and

(74) $${\hat{\sigma }}_{Y}^{2}=\sum_{\nu \neq 0}c_{\nu }^{2}.$$

The fitting routine calculates the root-mean-square residual

(75) $${\mathrm{RMSE}}_{\mathrm{fit}}=\sqrt{\frac{1}{N}\sum_{n=1}^{N}{\left[Y^{\left(n\right)}-{\hat{Y}}^{\left(n\right)}\right]}^{2}},$$

together with the condition number of the orthonormal regression matrix. These quantities were used as numerical diagnostics of the fitted expansions. Across the 32 beta-distribution cases, the full-sample fitting RMSE ranged from 0.0270 to 0.8404, with a median of 0.4082. When normalized by the standard deviation of the corresponding direct-model response, the full-sample RMSE ranged from 0.0266 to 0.1843, with a median of 0.1341. The corresponding training coefficients of determination ranged from $R^{2}=0.9660$ to $R^{2}=0.9993$, with a median of $R^{2}=0.9820$, and the condition numbers of the full 4000×969 regression matrices ranged from 3.27 to 9.60, with a median of 5.40.

Surrogate accuracy was additionally assessed using an 80/20 hold-out procedure for each of the 32 beta-distribution cases. From the 4000 available realizations, 3200 were used to refit the same 969-term third-order PCE, while the remaining 800 realizations were withheld from coefficient estimation and used only for out-of-sample prediction. Across the 32 cases, the hold-out coefficient of determination ranged from $R^{2}=0.9168$ to $R^{2}=0.9986$, with a median of $R^{2}=0.9568$. The validation RMSE normalized by the response standard deviation ranged from 0.0380 to 0.2883, with a median of 0.2077, whereas normalization by the observed validation-response range gave values from 0.00486 to 0.0449. The condition numbers of the 3200-sample training-subset regression matrices ranged from 4.01 to 12.47, with a median of 6.56. These diagnostics indicate generally good out-of-sample agreement and well-conditioned regression systems, while also showing reduced surrogate accuracy for several strongly skewed input-distribution cases. Accordingly, the Sobol indices are interpreted as sensitivity estimates obtained from an approximate third-order PCE surrogate rather than as exact indices of the underlying reduced-order model.

Because the Sobol indices used in the subsequent sensitivity analysis are obtained directly from the PCE coefficients, their robustness to the surrogate fitting procedure was assessed separately. For each of the 32 input-distribution cases, first- and total-order Sobol indices obtained from the full 4000-realization PCE were compared with those obtained after refitting the same third-order basis using only the 3200-realization training subset of the 80/20 hold-out analysis. Across all cases, the maximum absolute change in any first-order Sobol index ranged from $5.64\times 10^{-4}$ to $6.82\times 10^{-3}$, with a median of $2.06\times 10^{-3}$. The corresponding correlations between the full-sample and hold-out-refit first-order Sobol vectors ranged from 0.999607 to 0.999998. For the total-order indices, the maximum absolute changes ranged from $3.83\times 10^{-4}$ to $6.05\times 10^{-3}$, with a median of $1.99\times 10^{-3}$, and the corresponding correlations ranged from 0.999696 to 0.999999. The highest-ranked first-order and total-order sensitivity variables were unchanged in all 32 distribution cases. These results indicate that the reported sensitivity rankings and magnitudes are insensitive to the 20% reduction in fitting data used for the hold-out assessment.

### 6.4. Sobol Sensitivity Analysis

Variance-based global sensitivity analysis was performed using Sobol indices derived from the fitted Polynomial Chaos Expansions. The indices quantify the fraction of the surrogate-response variance associated with each uncertain input and with interactions among the inputs. They were calculated separately for each scalar response and each of the 32 beta-distribution shape cases.

For a response *Y*, the first-order Sobol index associated with input $X_{i}$ is

(76) $$S_{i}=\frac{{\mathrm{Var}}_{X_{i}}\left[E\left(Y|X_{i}\right)\right]}{\mathrm{Var}(Y)}.$$

The first-order index represents the contribution of $X_{i}$ acting alone, excluding interactions with the other inputs. The corresponding total-order index is

(77) $$S_{T_{i}}=1-\frac{{\mathrm{Var}}_{X_{∼i}}\left[E\left(Y|X_{∼i}\right)\right]}{\mathrm{Var}(Y)},$$

where $X_{∼i}$ denotes all uncertain inputs except $X_{i}$. The total-order index includes both the individual contribution of $X_{i}$ and all interaction terms involving that input.

Because the Jacobi PCE basis is orthonormal, the Sobol indices were calculated directly from the squared expansion coefficients. If ν denotes a PCE multi-index and $c_{\nu }$ is its coefficient, the first-order index is

(78) $$S_{i}=\frac{\sum_{\begin{array}{c} \nu :\;\nu_{i}>0\\ \nu_{j}=0,\;j\neq i \end{array}}c_{\nu }^{2}}{\sum_{\nu \neq 0}c_{\nu }^{2}},$$

whereas the total-order index is

(79) $$S_{T_{i}}=\frac{\sum_{\nu :\;\nu_{i}>0}c_{\nu }^{2}}{\sum_{\nu \neq 0}c_{\nu }^{2}}.$$

The computational implementation also calculates pairwise second-order indices from basis terms containing exactly two active inputs. Any remaining variance contribution is assigned to interactions involving three or more inputs.

For each beta-shape case, the indices represent sensitivity conditional on the corresponding common pair $\left(\alpha_{k},\beta_{k}\right)$. The sensitivity plot reported for $FoS_{\min,\mathrm{struct}}$ presents the arithmetic mean of the 32 case-specific first-order indices. This across-case mean is used as a descriptive distributional-robustness summary and should not be interpreted as a Sobol decomposition of an empirically established mixture distribution. Variation in the sensitivity rankings among the individual shape cases provides additional information regarding dependence on the assumed marginal distribution shape.

The resulting indices rank the uncertain model inputs according to their contributions to the variance predicted by the reduced-order surrogate. Accordingly, they can be used to identify parameters that merit improved measurement, tighter design control, additional experimental characterization, or higher-fidelity modeling. They should not be interpreted as experimentally validated causal measures of structural reliability or clinical outcome.

Each realization was also classified according to the response having the smallest calculated factor of safety among

(80) $$\left\{FoS_{\mathrm{buckling}},\;FoS_{\mathrm{combined}},\;FoS_{\mathrm{weld}},\;FoS_{\mathrm{pin}}\right\}.$$

This classification includes combined beam–column response, global buckling, weld loading, and halo-pin loading. Conditional tipping is excluded from this structural-mode classification and reported separately. Because the prescribed acceptance thresholds differ among the structural response modes, the resulting governing-mode plot identifies the smallest raw factor of safety, not necessarily the smallest factor of safety normalized by its required threshold. Actual limit-state violations were classified separately using the individual criteria in Equation (64).

## 7. Results and Discussion

The uncertainty-propagation analysis produced structural, operational, and clinical surrogate response quantities for the reduced-order halo-gravity traction wheelchair model. The evaluated responses include combined beam–column behavior, elastic buckling, weld loading, scenario-based tipping, halo-pin load concentration, delivered traction force, and an idealized clinical response metric. These quantities provide a screening-level description of the model behavior over the prescribed input bounds and alternative beta-distribution shape cases.

The analysis propagates patient-related loading, structural geometry, fabrication efficiency, support-polygon geometry, halo-pin load-transfer parameters, and mobility-related acceleration through the coupled clinical–structural model. The resulting distributions characterize the central tendency and dispersion of the predicted responses conditional on the adopted reduced-order equations, input ranges, independence assumption, and beta-distribution shapes. They should not be interpreted as empirically established population distributions or as experimentally validated measures of device reliability.

The propagated responses illustrate the interaction between patient-related loading and the mechanical configuration of the device. Patient weight and traction ratio determine the prescribed traction force, while structural section properties, load eccentricities, fabrication efficiency, support-polygon dimensions, rear-support and weight-stack locations, vertical carriage acceleration, and halo-pin load-sharing parameters influence the individual response measures. The relative importance of these inputs varies with the response quantity and the assumed beta-distribution shape.

The following subsections examine the descriptive response distributions, the response mode associated with the smallest calculated factor of safety, and the PCE-based sensitivity rankings. Limit-state-violation estimates are interpreted conditionally within the prescribed uncertainty models. Particular attention is given to the combined beam–column, halo-pin, and conditional tipping responses and to the limitations of drawing safety or clinical conclusions from the present unvalidated reduced-order framework.

### 7.1. Structural and Operational Screening Results

The coupled clinical–structural model was evaluated using 4000 Latin Hypercube realizations for each of the 32 bounded beta-distribution shape cases. The resulting response distributions describe the behavior of the reduced-order model over the prescribed input bounds and distributional-shape assumptions. They are conditional engineering screening results rather than empirically established population distributions.

For each realization, the descriptive minimum factor of safety was calculated as

(81) $$FoS_{\min,\mathrm{struct}}=\min\left\{FoS_{\mathrm{buckling}},\;FoS_{\mathrm{combined}},\;FoS_{\mathrm{weld}},\;FoS_{\mathrm{pin}}\right\}.$$

This quantity identifies the structural or pin-interface response having the smallest raw factor of safety. Because the required acceptance threshold is 2.0 for buckling and 1.0 for the other three responses, $FoS_{\min,\mathrm{struct}}$ is interpreted together with the individual criteria defined in Equation (64). Conditional tipping is excluded from this minimum and evaluated separately.

Table 5 reports the mean and the 5th, 50th, and 95th percentiles of the aggregate responses. The values were obtained by concatenating the valid realizations from the 32 alternative beta-shape cases with equal case weighting. They are reported as descriptive distributional-robustness summaries.

The aggregate results show that the combined beam–column and halo-pin responses have substantially lower central factors of safety than the buckling and weld responses. The conditional tipping envelope is reported separately because it corresponds to prescribed stop, drop, and slope conditions rather than an ordinary-use event probability. These differences illustrate that each predicted response depends on its governing mechanical relationship and assumed input distributions. They should not be interpreted as experimental evidence of device safety.

**Figure 2:**
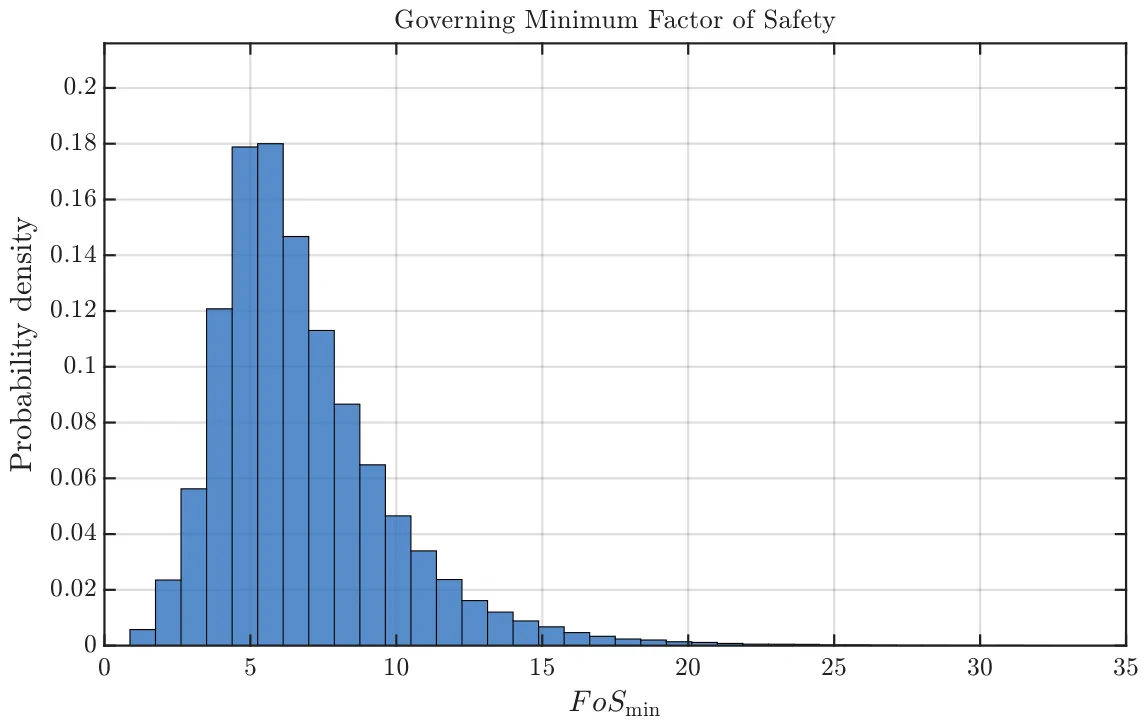
Aggregate probability-density representation of the descriptive structural and pin-interface minimum factor of safety across the 32 beta-distribution shape cases.

Figure 2 presents the aggregate distribution of $FoS_{\min,\mathrm{struct}}$. The mean is approximately 6.81, and the 5th–95th percentile interval extends from approximately 3.14 to 12.51. The distribution reflects switching among the four structural and pin-interface response modes that produce the smallest raw factor of safety in different realizations. It should therefore be interpreted together with the individual response distributions and their mode-specific acceptance criteria.

The combined beam–column response is lower than the Euler buckling and weld responses over much of the investigated space because it includes direct compression, eccentric bending, and elastic moment magnification. The relatively low pin response arises from uncertainty in the engineering pin–skull screening load, pin-load amplification, load-sharing efficiency, number of active pins, and prescribed traction force.

**Figure 3:**
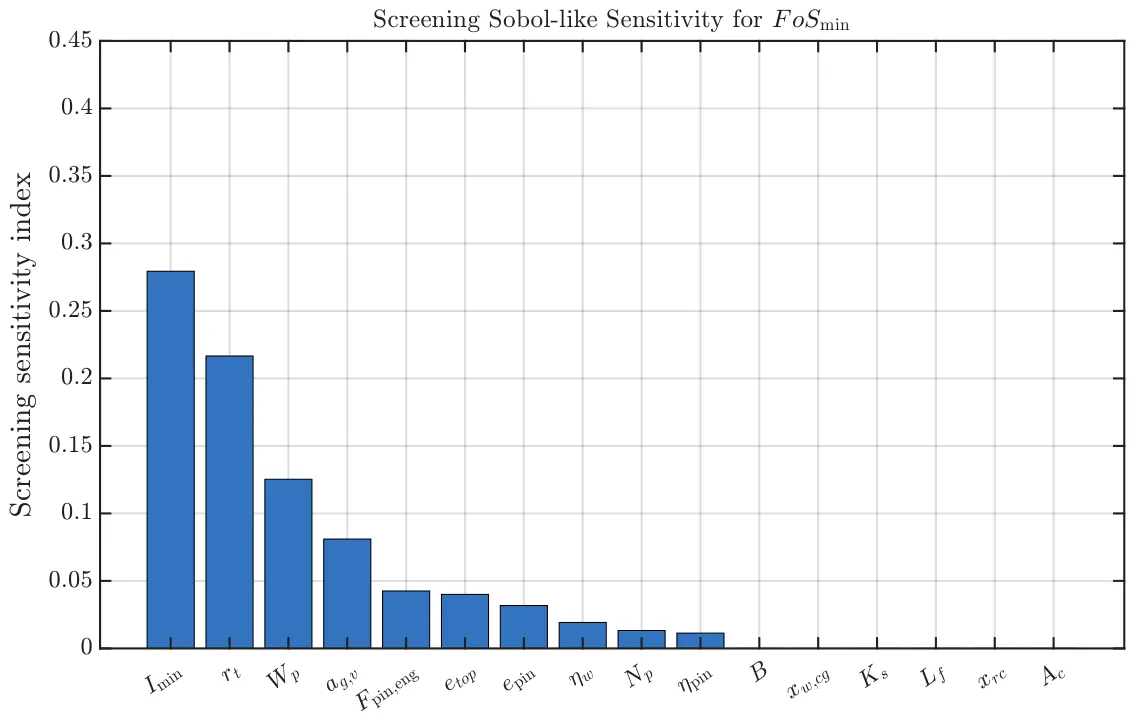
Across-case mean first-order Sobol sensitivity indices for the descriptive minimum factor of safety.

The first-order indices shown in Figure 3 are arithmetic means of the indices calculated separately for the 32 beta-shape cases. The largest mean indices are associated primarily with the halo-pin subsystem and prescribed traction loading. The engineering pin–skull screening load, pin-load amplification factor, number of active pins, traction ratio, and pin load-sharing efficiency influence $FoS_{\min,\mathrm{struct}}$ mainly by changing the halo-pin factor of safety and the frequency with which the pin response becomes the smallest raw factor of safety. Structural-section and support-geometry variables influence other response modes but have smaller mean first-order contributions to the aggregate $FoS_{\min,\mathrm{struct}}$ surrogate over the investigated input space.

Because the plotted indices are averages across alternative distributional shapes, they are interpreted as screening-level sensitivity summaries. Case-specific rankings may differ from the across-case mean.

**Figure 4:**
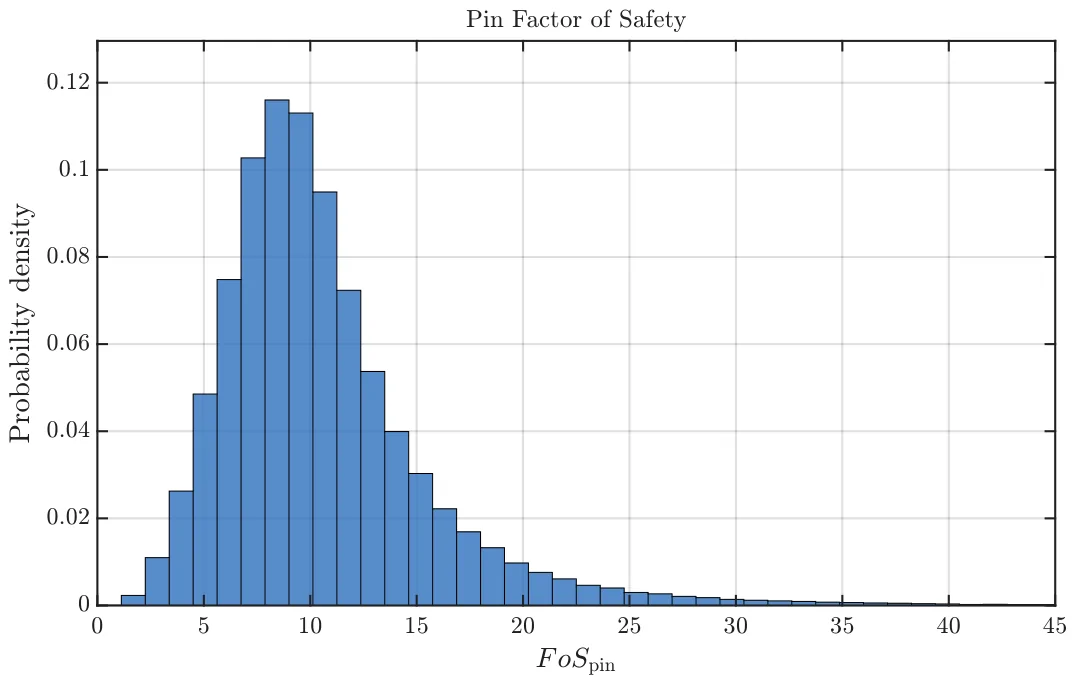
Aggregate probability-density representation of the halo-pin engineering screening factor across the 32 beta-shape cases.

The halo-pin screening-factor distribution shown in Figure 4 has a mean of approximately 10.65 and a 5th–95th percentile interval of approximately 4.65–19.97. This response is conditional on the simplified load-sharing equation and the adopted 50–175 lb engineering interface screening range. $FoS_{\mathrm{pin}}$ should not be interpreted as a clinically validated pediatric pin-safety factor.

**Figure 5:**
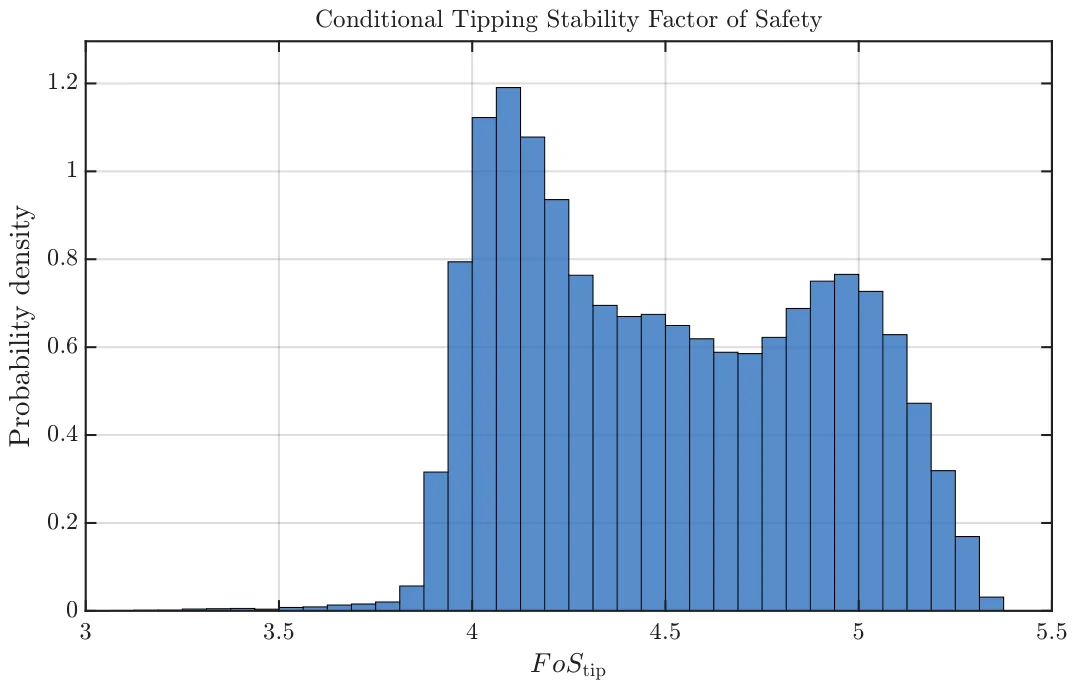
Aggregate probability-density representation of the conditional tipping-stability envelope across the 32 beta-shape cases.

Figure 5 presents the distribution of $FoS_{\mathrm{tip}}=\min\left(FoS_{f},FoS_{\ell },FoS_{r}\right)$ under the prescribed forward-stop, one-sided curb-drop, and uphill-slope conditions. The mean is approximately 4.51, and the 5th–95th percentile interval extends from approximately 3.97 to 5.15 (Table 6). Variation in the conditional envelope arises from patient weight, traction ratio, rear-support location, guided weight-stack location, front wheelbase, and wheel-contact track. The stop deceleration, drop height, and slope are fixed at 0.25g, 2 in, and 10 degrees, respectively. The conditional screening criterion is $FoS_{\mathrm{tip}}<1.5$; it is not an estimate of accident probability.

The moderate distributional spread reflects variation in the component weights and support-polygon geometry. The calculation remains a quasi-static support-polygon screening model and does not resolve transient wheel–ground contact, caster motion, curb-impact dynamics, structural flexibility during impact, or caregiver-applied forces. The distribution therefore does not constitute experimental verification of dynamic anti-tipping performance.

**Figure 6:**
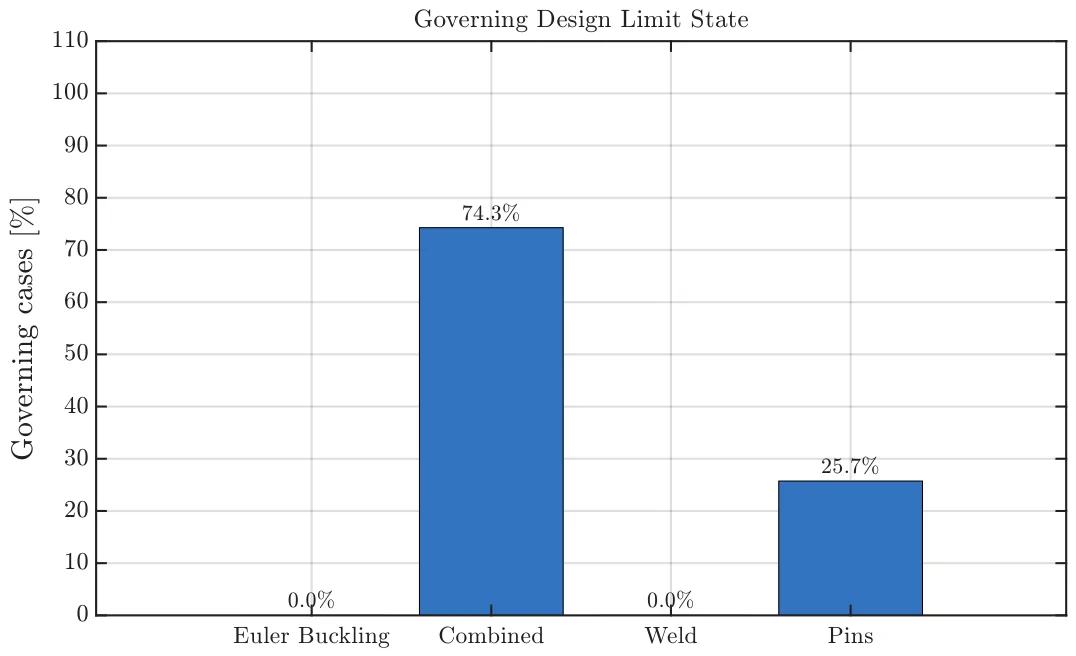
Distribution of the structural or pin-interface response associated with the smallest raw factor of safety across the uncertainty realizations.

Figure 6 classifies each realization according to the structural or pin-interface response having the smallest raw factor of safety. The combined beam–column and halo-pin responses are the most frequent minimum-factor modes, while Euler buckling and weld loading are less frequently identified. Tipping is intentionally excluded because it is a conditional scenario screen. Because the prescribed structural acceptance thresholds differ among these modes, the figure should not be interpreted as a classification based on normalized limit-state margin.

**Figure 7:**
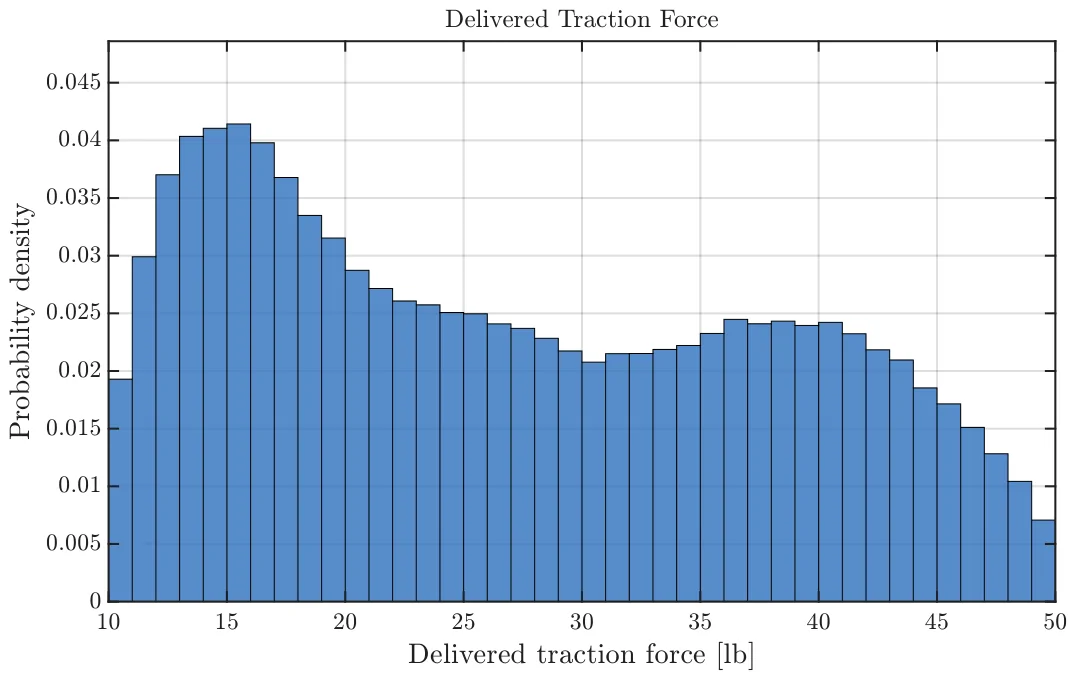
Aggregate probability-density representation of the delivered traction force across the 32 beta-shape cases.

The delivered traction-force distribution shown in Figure 7 has a mean of approximately 27.09 lb and a 5th–95th percentile interval of approximately 12.02–45.72 lb. In the present formulation, the reported delivered traction is equal to the prescribed traction force, $F_{t,d}=F_{t}$. The fixed pulley efficiency is used only to calculate the guided weight-stack load and associated internal structural and inertial loads.

The clinical surrogate is calculated from

(82) $$\Delta L_{s}=\frac{F_{t,d}}{K_{s}},$$

and

(83) $$\eta_{c}=\frac{\Delta L_{s}}{\Delta L_{\mathrm{target}}},\;\Delta L_{\mathrm{target}}=1.5\;\mathrm{in}.$$

**Figure 8:**
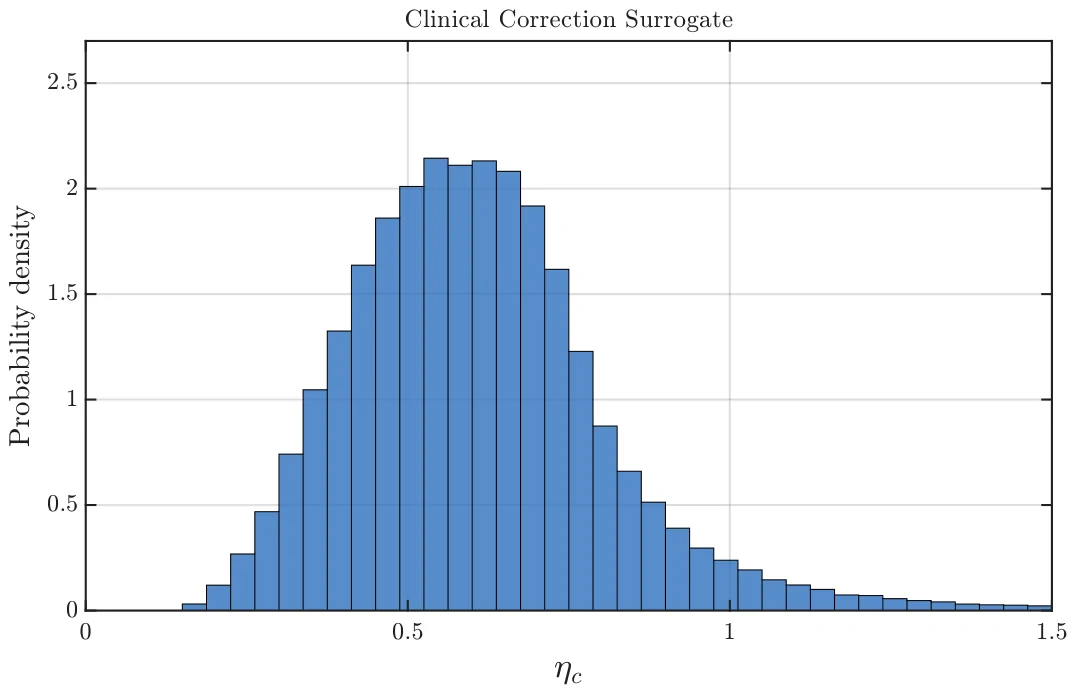
Aggregate probability-density representation of the normalized clinical surrogate response across the 32 beta-shape cases.

The aggregate $\eta_{c}$ distribution shown in Figure 8 has a mean of approximately 0.612 and a 5th–95th percentile interval of approximately 0.324–0.970. This quantity is an idealized linear-stiffness surrogate and should not be interpreted as a predicted percentage of clinical correction or as a patient-specific treatment outcome.

Overall, the results identify the combined beam–column and halo-pin calculations as the most influential components of the primary structural screening framework, while the separate tipping calculations quantify margins under three prescribed mobility scenarios. The reported response distributions and sensitivity indices provide information for prioritizing subsequent model refinement and testing, but they do not establish structural certification, clinical safety, or operational reliability of the prototype.

### 7.2. Design Interpretation and Future Structural Refinement

The uncertainty-propagation results provide information beyond a nominal deterministic calculation by identifying the inputs that control the individual screening responses and the variation in those responses over the prescribed uncertainty space. However, the magnitude of the calculated factors of safety should not be interpreted as experimental confirmation of structural adequacy. The results remain conditional on the reduced-order load paths, geometric assumptions, input bounds, and quasi-static representation of mobility-related loading.

Future development should initially focus on validating the assumed load paths and response models rather than simply increasing nominal component strength. Priority activities include measured prototype load testing of the vertical support and welded frame, verification of the effective section properties, measurement of pulley and carriage loads, weld inspection and local stress assessment, and static and dynamic tipping tests. Instrumented tests involving braking, turning, ramps, thresholds, and representative floor transitions would help determine the accelerations transmitted to the guided weight carriage and assess whether the adopted vertical $a_{g,v}=0$–1.0 beam–column screening interval adequately represents routine operation. Separate wheelchair tests should measure horizontal deceleration during attendant stopping and validate the prescribed 0.25g forward-tipping case.

The tipping analysis indicates that the front wheelbase, wheel-contact track, rear-support location, and component center-of-gravity positions should be treated as coupled design considerations. A longer front wheelbase increases the restoring lever arm during stopping, a wider track increases lateral stability during a one-sided drop, and greater rear-support extension improves the uphill rearward margin. Locating the guided weight assembly low and within the support polygon is beneficial in all three cases. These changes should nevertheless be evaluated together with maneuverability, doorway clearance, caregiver access, and the risk of interference between the rear supports and the surrounding environment.

The halo-pin results similarly indicate that uncertainty in the engineering interface screening load, pin-load amplification, load-sharing efficiency, and number of active pins strongly influences the corresponding reduced-order response. These findings primarily identify the need for improved biomechanical characterization; they should not be used to prescribe pin number, insertion torque, or clinical loading without appropriate clinical evidence.

Alternative structural materials, including fiber-reinforced composites, may eventually offer reductions in structural mass because of their high specific stiffness and strength. Such a substitution should not be inferred directly from the present analysis, which models an isotropic steel support and welded connections. A composite redesign would require a separate assessment of laminate anisotropy, joint and attachment design, local bearing and delamination, impact tolerance, environmental durability, damage inspectability, manufacturability, and applicable medical-device requirements. Material substitution should therefore follow, rather than precede, experimental validation of the governing loads and structural response of the current prototype.

## 8. Limitations

The propagated distributions, sensitivity indices, and limit-state estimates are conditional on the adopted reduced-order equations, prescribed bounds, 32 alternative beta-shape specifications, and independent-input assumption. The beta distributions were not fitted to population data, and the aggregate statistics describe an equal-weight distributional-robustness exercise rather than population-level clinical probabilities.

The structural model has not yet been validated through dedicated full-system prototype load tests, finite-element analysis, or multibody simulation. The effective section properties are sampled independently, the weld calculation is a nominal throat-stress screen rather than a local fatigue-life assessment, and the tipping model is quasi-static. Transient wheel–ground contact, caster rotation, curb impacts, caregiver-applied forces, structural flexibility, and the detailed acceleration history of the carriage are not resolved.

The tipping calculations prescribe a 0.25g abrupt stop, a 2 in one-sided drop, and a 10-degree uphill slope. Their occurrence probabilities are not modeled; therefore, the resulting $FoS_{\mathrm{tip}}$ distribution is a conditional stability envelope rather than an ordinary-use accident or failure probability. The wheelbase and track ranges are representative of a standard hospital wheelchair rather than measurements of the completed prototype, and the component center-of-gravity heights and lateral offsets are reduced-order assumptions. Dynamic impact at ground contact after a curb drop is outside the present formulation.

The halo-pin formulation is an engineering load-transfer surrogate and does not constitute a clinically validated pediatric pin–skull failure criterion. It omits patient-specific skull thickness and bone quality, insertion details, local contact mechanics, biological adaptation, loosening, and combined loading interactions. Similarly, the spinal-elongation response is an idealized clinical surrogate and must not be interpreted as a patient-specific prediction of correction or treatment outcome.

Finally, the third-order PCE surrogates were assessed using their training-set fit and coefficient-derived statistics but were not validated against a separate independent test set. Future work should therefore include prototype static and dynamic testing, higher-fidelity structural models, independent surrogate validation, correlated-input studies, weld and fatigue characterization, and improved pediatric halo-interface biomechanics before the framework is used for certification or clinical decision-making.

## 9. Conclusions

A preliminary coupled clinical–structural uncertainty-screening framework was developed for a prototype mobile halo-gravity traction wheelchair. The framework combines reduced-order models of traction delivery, eccentric beam–column response, elastic buckling, welded-joint loading, scenario-based wheelchair tipping, halo-pin load concentration, and an idealized clinical response surrogate. Sixteen patient-related, operational, geometric, fabrication, and halo-interface inputs were propagated using Latin Hypercube Sampling with bounded beta distributions.

Thirty-two common beta-distribution shape cases were examined, with 4000 model realizations evaluated for each case. Separate third-order Jacobi Polynomial Chaos Expansions were fitted to the scalar responses using an 969-term total-order basis, and the resulting coefficients were used to calculate response moments and variance-based sensitivity indices. The alternative distributional-shape cases were used to examine the dependence of the predicted responses on the assumed marginal distribution shapes rather than to represent empirically fitted population distributions.

Across the equal-weight aggregate of the 32 shape cases, the descriptive structural and pin-interface minimum factor of safety had a mean of approximately 6.81 and a 5th–95th percentile interval of approximately 3.14–12.51. The combined beam–column response and halo-pin screening response produced lower central factors of safety than the Euler buckling and weld responses. The conditional tipping envelope had a mean of approximately 4.51 and a 5th–95th percentile interval of approximately 3.97–5.15. These values apply only to the prescribed 0.25g stop, 2 in one-sided drop, and 10-degree uphill slope and do not represent the probability of a tipping accident.

The averaged first-order sensitivity indices for the descriptive minimum factor of safety were influenced primarily by the halo-pin screening parameters and prescribed traction loading. These results identify the engineering pin–skull screening load, pin-load amplification, number of active pins, traction ratio, and pin load-sharing efficiency as important inputs within the present reduced-order formulation. The sensitivity rankings are conditional on the adopted model, bounds, independence assumption, PCE approximation, and beta-distribution shapes and should not be interpreted as experimentally validated causal measures.

The analysis also demonstrates the value of treating wheelchair stability as three explicit conditional scenarios rather than as a fictitious horizontal carriage-acceleration limit state. The forward-stop margin is governed by the front-caster lever arm and component center-of-gravity heights; the one-sided drop margin depends strongly on track width and drop geometry; and the uphill rearward margin depends on the rear support and longitudinal mass distribution. Dedicated static and dynamic tests are needed to assess braking, turning, ramps, thresholds, curb negotiation, patient movement, caregiver handling, and transient wheel–ground interaction.

The reported factors of safety, limit-state indicators, response distributions, and sensitivity indices are computational screening results. The reduced-order model has not been validated through dedicated full-system prototype load testing or higher-fidelity finite-element and multibody simulation. The halo-pin formulation is not a clinically validated pediatric pin–skull failure criterion, and the clinical response metric is not a patient-specific biomechanical prediction. Consequently, the present results do not certify structural safety, establish clinical efficacy, or provide population-level failure probabilities.

The principal value of the framework is its ability to identify influential model inputs, compare interacting response modes, and prioritize subsequent engineering work. Future development should include prototype static-load testing, measurement of operational carriage accelerations, dynamic tipping evaluation, verification of structural section properties, weld inspection and fatigue characterization, improved halo-interface biomechanics, explicit treatment of correlated inputs, independent PCE validation, and higher-fidelity structural modeling. These activities are necessary before the framework can support conclusions regarding clinical implementation or device certification.

## Figures and Tables

**Table 1:** Representative dimensions of the standard hospital wheelchair used in the tipping analysis. Envelope dimensions describe the complete chair, whereas the wheelbase and track define the ground-contact support polygon.

Dimension	Nominal Value	Model Treatment
Overall length with footrests	42 in	Descriptive
Overall width	26 in	Descriptive
Overall height	36 in	Descriptive
Seat depth	16 in	Fixed
Seat-to-floor height	19 in	Representative
Rear-wheel diameter	24 in	Fixed
Front-caster diameter	8 in	Fixed
Rear axle to front-caster contact, $L_{f}$	18 in	17–20 in in UQ
Rear-wheel contact-center track, *B*	24 in	23–25 in in UQ

**Table 2:** Deterministic tipping checks for the representative wheelchair and nominal mass distribution.

Prescribed Scenario	Scenario Value	Factor of Safety
Abrupt forward stop	$a_{\mathrm{stop}}=0.25g$	$FoS_{f}=4.67$
One-sided lateral drop	d=2 in	$FoS_{\ell }=7.37$
Uphill rearward check	$\theta =10^{\circ }$	$FoS_{r}=5.61$
Severe one-sided drop	d=6 in	$FoS_{\ell }=2.39$
Runaway stopping illustration	10 m/s stopped in 1 s	$FoS_{f}=1.14$

**Table 3:** Uncertain input variables and adopted bounds used in the coupled clinical–structural uncertainty quantification framework.

Variable	Description	Lower	Upper
$W_{p}$	Patient body weight [lb]	50	100
$r_{t}$	Traction-to-body-weight ratio	0.20	0.50
$K_{s}$	Effective spinal stiffness surrogate [lb/in]	15	45
$N_{p}$	Number of active halo pins	4	8
$F_{\mathrm{pin},\mathrm{eng}}$	Engineering pin–skull screening load [lb]	50	175
$e_{\mathrm{pin}}$	Pin-load amplification factor	1.0	3.0
$\eta_{\mathrm{pin}}$	Halo-pin load-sharing efficiency	0.50	1.00
$A_{c}$	Effective vertical-support area [in.^2^]	1.0	1.8
$I_{\min}$	Weak-axis second moment of area [in.^4^]	0.05	0.25
$e_{\mathrm{top}}$	Upper-load-line eccentricity [in.]	12	20
$\eta_{w}$	Weld/fabrication efficiency factor	0.70	1.00
$x_{rc}$	Rear-caster support location [in.]	18	30
$x_{w,\mathrm{cg}}$	Weight-stack center-of-gravity location [in.]	12	23
$a_{g,v}$	Adverse vertical carriage acceleration normalized by *g*	0.00	1.00
$L_{f}$	Rear axle to front-caster contact [in.]	17	20
*B*	Wheel-contact track [in.]	23	25

For each distributional-shape case, the continuous inputs were sampled independently from bounded beta distributions over the listed intervals. The latent sample for $N_{p}$ was mapped to [4,8] and rounded to the nearest integer before model evaluation.

**Table 4:** Common beta-distribution shape parameters used in the 32 distributional-shape cases.

Case	$\alpha_{k}$	$\beta_{k}$	Case	$\alpha_{k}$	$\beta_{k}$
1	0.5	0.5	17	1.5	4.0
2	0.8	0.8	18	4.0	1.5
3	1.0	1.0	19	2.0	8.0
4	1.5	1.5	20	8.0	2.0
5	2.0	2.0	21	0.6	1.5
6	3.0	3.0	22	1.5	0.6
7	5.0	5.0	23	0.8	5.0
8	8.0	8.0	24	5.0	0.8
9	0.5	2.0	25	1.2	2.5
10	2.0	0.5	26	2.5	1.2
11	1.0	3.0	27	1.5	6.0
12	3.0	1.0	28	6.0	1.5
13	2.0	5.0	29	3.0	8.0
14	5.0	2.0	30	8.0	3.0
15	0.8	3.0	31	0.7	4.0
16	3.0	0.8	32	4.0	0.7

**Table 5:** Descriptive summary statistics for the structural and clinical responses obtained across the 32 beta-distribution shape cases.

Response	Mean	SD	P05	P50	P95
$FoS_{\min,\mathrm{struct}}$	6.8117	3.0750	3.1405	6.1563	12.5143
$FoS_{\mathrm{combined}}$	7.8029	4.3158	3.3029	6.6282	16.1253
$FoS_{\mathrm{pin}}$	10.6517	5.3628	4.6502	9.6072	19.9696
$FoS_{\mathrm{buckling}}$	63.1974	31.8821	28.7263	54.8112	124.9865
$FoS_{\mathrm{weld}}$	112.1109	61.5527	45.6089	92.9158	233.4146
$F_{t,d}$ [lbf]	27.0919	11.0874	12.0206	25.6673	45.7197
$\eta_{c}$	0.6117	0.2074	0.3245	0.5935	0.9698

Each response contains 128,000 valid realizations obtained by pooling 4000 realizations from each of the 32 alternative beta-distribution shape cases. These statistics are descriptive distributional-robustness summaries and should not be interpreted as population-level clinical probabilities. Conditional wheelchair-tipping results are reported separately because the prescribed stopping, one-sided curb-drop, and uphill-slope conditions are scenario-based stability screens rather than ordinary-use probabilistic failure modes.

**Table 6:** Descriptive statistics for the conditional tipping-stability envelope across the 32 beta-distribution shape cases.

Response	Mean	SD	P05	P50	P95
$FoS_{\mathrm{tip}}$	4.5063	0.3972	3.9684	4.4640	5.1484

## Data Availability

The data presented in this study are available from the corresponding author upon reasonable request.

## Nomenclature

$A_{c}$	Effective cross-sectional area of the vertical support
$A_{w}$	Effective weld-throat area
$a_{g,v}$	Adverse vertical guided-carriage acceleration normalized by gravitational acceleration
$a_{\mathrm{stop}}$	Prescribed wheelchair deceleration in the forward-tipping scenario
*B*	Wheel-contact track used in the lateral-tipping model
B(α,β)	Beta function
*c*	Distance from the neutral axis to the extreme fiber
$c_{\nu }$	PCE coefficient associated with multi-index ν
*d*	One-sided wheel-drop height in the lateral-tipping scenario
*E*	Young’s modulus
$e_{\mathrm{pin}}$	Pin-load amplification factor
$e_{\mathrm{top}}$	Horizontal offset from vertical-member centroid to upper load line
$F_{d,v}$	Adverse vertical inertial force acting on the guided carriage
$F_{t}$	Prescribed traction force
$F_{t,d}$	Reported delivered traction force
$F_{w}$	Guided weight-stack load
$F_{\mathrm{pin},\mathrm{avg}}$	Average traction-induced load per active halo pin
$F_{\mathrm{pin},\max}$	Estimated maximum traction-induced load on an individual halo pin
$F_{\mathrm{pin},\mathrm{eng}}$	Engineering pin–skull interface screening load
$FoS_{\mathrm{buckling}}$	Euler buckling factor of safety
$FoS_{\mathrm{combined}}$	Combined axial-compression and bending factor of safety
$FoS_{\mathrm{fatigue}}$	Diagnostic fatigue comparison factor
$FoS_{f}$	Conditional forward-stop tipping factor of safety
$FoS_{\ell }$	Conditional one-sided-drop lateral tipping factor of safety
$FoS_{\min,\mathrm{struct}}$	Minimum of the four structural and pin-interface screening factors
$FoS_{\mathrm{pin}}$	Halo-pin engineering screening factor
$FoS_{r}$	Conditional uphill rearward-tipping factor of safety
$FoS_{\mathrm{tip}}$	Minimum of the forward, lateral, and rearward conditional tipping factors
$FoS_{\mathrm{weld}}$	Weld factor of safety
$g_{j}\left(X\right)$	Limit-state function for response mode *j*
$h_{w}$	Effective height of the guided weight carriage
$h_{\mathrm{weld}}$	Weld size used in the effective throat-area calculation
$I_{f}$	Binary engineering limit-state-violation indicator
$I_{\min}$	Weak-axis second moment of area of the vertical support
*K*	Effective-length factor for column buckling
$K_{s}$	Effective linear spinal-stiffness surrogate
$L_{c}$	Unsupported length of the vertical support
$L_{f}$	Rear-axle-to-front-caster contact distance
$L_{\mathrm{weld}}$	Effective weld length
$M_{0}$	First-order eccentric bending moment
$M_{\mathrm{amp}}$	Elastically magnified beam–column moment
$M_{R,q}$	Restoring moment for tipping scenario q∈{f,ℓ,r}
$M_{O,q}$	Overturning moment for tipping scenario q∈{f,ℓ,r}
*N*	Number of LHS realizations in each beta-shape case
$N_{\mathrm{basis}}$	Number of retained PCE basis terms
$N_{p}$	Number of active halo pins
$P_{\mathrm{cr}}$	Euler critical buckling load
$P_{\mathrm{eq}}$	Equivalent compressive load used in the structural model
${\hat{P}}_{f,k}$	Estimated conditional limit-state-violation probability for case *k*
p(ξ;α,β)	Beta probability-density function
$r_{t}$	Prescribed traction-to-body-weight ratio
$S_{i}$	First-order Sobol sensitivity index for input *i*
$S_{T_{i}}$	Total-order Sobol sensitivity index for input *i*
$W_{p}$	Patient body weight
$W_{\mathrm{sys}}$	Wheelchair and support-system weight
$W_{\mathrm{sys},\dim}$	Overall system width, when needed to distinguish it from weight
$H_{\mathrm{sys}}$	Overall system height
$x_{p,\mathrm{cg}}$	Longitudinal location of the patient center of gravity
$x_{f}$	Longitudinal location of the front-caster tipping axis
$x_{rc}$	Longitudinal location of the rear anti-tip support axis
$x_{\mathrm{sys},\mathrm{cg}}$	Longitudinal location of the support-system center of gravity
$x_{w,\mathrm{cg}}$	Longitudinal location of the guided weight-stack center of gravity
X	Complete uncertain input vector
$X_{c}$	Patient-related uncertain input vector
$X_{d}$	Device-, fabrication-, and operation-related uncertain input vector
$X_{∼i}$	Complete input vector excluding $X_{i}$
Y	Model response vector
$\Delta L_{s}$	Spinal-displacement surrogate
$\Delta L_{\mathrm{target}}$	Fixed normalization displacement for the clinical surrogate
$\alpha_{k},\beta_{k}$	Common beta-distribution shape parameters for case *k*
ν	Multivariate PCE basis multi-index
$\eta_{c}$	Normalized clinical-response surrogate
$\eta_{p}$	Fixed pulley-efficiency factor
$\eta_{\mathrm{pin}}$	Halo-pin load-sharing efficiency
$\eta_{w}$	Weld/fabrication efficiency factor
ϕ	Wheelchair roll angle produced by the prescribed one-sided drop
$\sigma_{\mathrm{alt}}$	Representative alternating stress used in fatigue screening
$\sigma_{c}$	Average axial compressive stress
$\sigma_{\mathrm{endurance}}$	Assumed material endurance strength
$\sigma_{\max}$	Extreme-fiber combined axial and bending stress
$\sigma_{y}$	Material yield strength
$\tau_{\mathrm{allow}}$	Nominal allowable weld shear stress
$\tau_{w}$	Average weld-throat shear stress
θ	Prescribed uphill slope angle in the rearward-tipping scenario
$\Psi_{\nu }$	Multivariate orthonormal PCE basis function
$\xi_{i}$	Normalized beta-distributed input variable
